# Multi-omics in exploring the pathophysiology of diabetic retinopathy

**DOI:** 10.3389/fcell.2024.1500474

**Published:** 2024-12-11

**Authors:** Xinlu Li, XiaoJing Dong, Wen Zhang, Zhizhou Shi, Zhongjian Liu, Yalian Sa, Li Li, Ninghua Ni, Yan Mei

**Affiliations:** ^1^ Faculty of Life Science and Technology, Kunming University of Science and Technology, Kunming, China; ^2^ Department of Ophthalmology, The Affiliated Hospital of Kunming University of Science and Technology, Kunming, China; ^3^ Department of Ophthalmology, The First People’s Hospital of Yunnan Province, Kunming, China; ^4^ Medical School, Kunming University of Science and Technology, Kunming, China; ^5^ Institute of Basic and Clinical Medicine, The First People’s Hospital of Yunnan Province, Kunming, China

**Keywords:** diabetic retinopathy (DR), multi-omics, single-cell RNA sequencing(scRNA-seq), transcriptomics, genomics, metabolomic, lipidomic

## Abstract

Diabetic retinopathy (DR) is a leading global cause of vision impairment, with its prevalence increasing alongside the rising rates of diabetes mellitus (DM). Despite the retina’s complex structure, the underlying pathology of DR remains incompletely understood. Single-cell RNA sequencing (scRNA-seq) and recent advancements in multi-omics analyses have revolutionized molecular profiling, enabling high-throughput analysis and comprehensive characterization of complex biological systems. This review highlights the significant contributions of scRNA-seq, in conjunction with other multi-omics technologies, to DR research. Integrated scRNA-seq and transcriptomic analyses have revealed novel insights into DR pathogenesis, including alternative transcription start site events, fluctuations in cell populations, altered gene expression profiles, and critical signaling pathways within retinal cells. Furthermore, by integrating scRNA-seq with genetic association studies and multi-omics analyses, researchers have identified novel biomarkers, susceptibility genes, and potential therapeutic targets for DR, emphasizing the importance of specific retinal cell types in disease progression. The integration of scRNA-seq with metabolomics has also been instrumental in identifying specific metabolites and dysregulated pathways associated with DR. It is highly conceivable that the continued synergy between scRNA-seq and other multi-omics approaches will accelerate the discovery of underlying mechanisms and the development of novel therapeutic interventions for DR.

## Graphical Abstract

**Figure:**
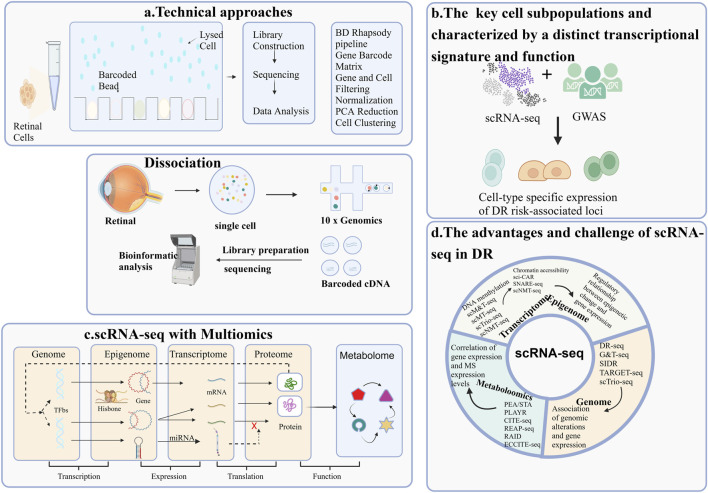
Overview of single-cell RNA sequencing (scRNA-seq) Applications in Diabetic Retinopathy Research. The application of single-cell RNA sequencing (scRNA-seq) methodologies in the study of diabetic retinopathy(DR) highlights four key areas: **(A)** The workflow of a typical scRNA-seq experiment; **(B)** The identification of key cell subpopulations, each characterized by a distinct transcriptional signature and function; **(C)** The integration of scRNA-seq with multi-omics approaches for DR research; **(D)** The advantages and challenges associated with scRNA-seq.

## 1 Introduction

Diabetic retinopathy (DR) is a leading cause of vision impairment and blindness among the working-age population globally (Bryl et al., 2022; Tan and Wong, 2022; Wurl et al., 2023). Projections indicate a substantial increase in the number of affected adults, with estimates reaching 129.84 million by 2030 and 160.50 million by 2045, posing significant societal and economic burdens (Ashraf et al., 2020; Yang et al., 2024; Yuan et al., 2024). The molecular mechanisms underlying DR encompass pathways such as inflammation, oxidative stress, renin-angiotensin system activation, and vascular endothelial growth factor (VEGF) signaling (Nouri et al., 2024). However, there remains a considerable gap in our understanding of the specific cellular alterations and their complex interactions in the disease’s progression. Emerging evidence suggests that abnormalities in cellular metabolic pathways, such as glucose, amino acid, and lipid metabolism, also play crucial roles in DR pathogenesis (Rohlenova et al., 2020; Yang Z et al., 2023). These metabolic disruptions lead to cellular stress, inflammation, and vascular damage, which are pivotal in disease development. Recognized increasingly as a neurodegenerative and neuroinflammatory disorder, DR involves intricate interactions among dysfunctional glial cells, neurons, and endothelial cells (ECs), highlighting its complexity (Llorian-Salvador et al., 2024).

Transcriptomic profiling of aqueous humor (AH), vitreous humor (VH), and retinal tissue has offered valuable insights into the global genomic alterations associated with DR pathogenesis, contributing to the identification of potential biomarkers and therapeutic targets (Wolf et al., 2023). However, the retina is a complex tissue composed of diverse cell types. Conventional transcriptomic profiles of DR, derived from heterogeneous cell populations, obscure crucial information about specific vulnerable cell types. Single-cell RNA sequencing (scRNA-seq) emerges as an unbiased and potent technique for characterizing distinct cell populations within complex tissues under both healthy and diseased conditions (Blanchard et al., 2022).

The exponential growth of scRNA-seq has dramatically enhanced our capacity to quantify ligand and receptor expression across diverse cell types, enabling the systematic elucidation of intercellular communication networks that underlie tissue function in both health and disease states (He et al., 2020; Ma et al., 2023).

Additionally, Xia et al. (2023) employed scRNA-seq in conjunction with genetic perturbation to precisely localize a specific pericyte subpopulation adjacent to pathological neovascularization tufts (Liu et al., 2022a). These findings underscore the potential of single-cell analyses to provide in-depth insights into distinct cell subpopulations and their roles in pathophysiological processes.

Multi-omics analyses (Fan and Pedersen, 2021; Guo et al., 2024) at the bulk retina level have been instrumental in providing a comprehensive understanding of cellular processes by integrating diverse molecular data, including mutations, mRNAs, proteins, and metabolites. Wang N et al. (2022) pioneered advanced methodologies for concurrent genomics and transcriptomics, leading to the identification of genes associated with DR. This finding was validated across independent cohorts and computational models, demonstrating the study’s robustness (Koh and Bäckhed, 2020).

The innovative approach employed in this research holds broad implications for understanding the complex genetic underpinnings of various diseases (Zhang and Zhao, 2016). This study also lays the foundation for developing novel therapeutic strategies for DR, with the potential to surpass current treatments such as laser surgery and intraocular injections.

In conclusion, integrating gene expression data from relevant cellular models with genetic association data has provided critical insights into the functional relevance of genetic risk factors for complex diseases such as DR. The identification of disease-associated differential gene expression and the utilization of expression quantitative trait loci based genome-wide association studies (GWAS) have been instrumental in elucidating potential causal genetic pathways underlying DR (Peng et al., 2023).

Identifying ligand-receptor interactions from scRNA-seq data involves integrating annotated relationships with statistical methods (Mao et al., 2024). This approach has spurred the development of single-cell multi-omics technologies, utilizing diverse experimental protocols such as mRNA-DNA methylation and mRNA-protein analysis to investigate cell type-specific gene regulation (Lv et al., 2022). Yao et al. (2022) employed scRNA-seq to elucidate EC heterogeneity and functional diversity. Their systematic analysis identified a unique EC cluster within the diabetic retina characterized by elevated inflammatory gene expression (Ma et al., 2023; Samanta et al., 2024). Therefore, single-cell multi-omics analysis offers a more comprehensive understanding of cell type-specific gene regulation than single-cell mono-omics approaches. This integrated approach significantly enhances our comprehension of the cellular and molecular landscape in DR, enabling the identification of affected cell types, elucidation of dysfunctional pathways, and discovery of potential therapeutic targets.

**FIGURE 1:**
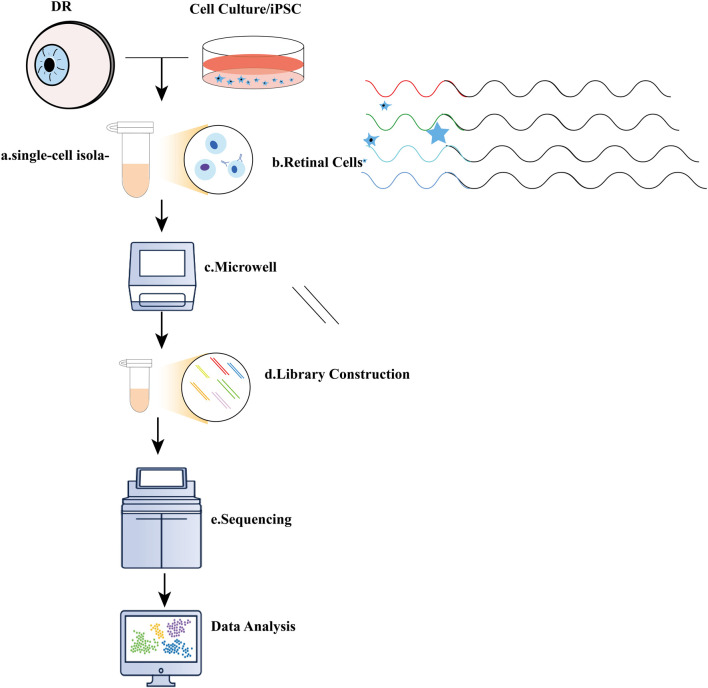
Illustration of single-cell RNA sequencing (scRNA-seq) experiments. **(A)** Typical single-cell RNA sequencing (scRNA-seq) workflow encompasses several key steps: a. single-cell isolation, which can be achieved through techniques such as micromanipulation, laser-capture microdissection, microfluidics, or fluorescence-activated cell sorting; **(B)**. Cell lysis; **(C)** Reverse transcription of mRNA into complementary DNA (cDNA); **(D)** Amplification of cDNA and library preparation; **(E)** Sequencing.

In this review, we explore the specific applications of scRNA-seq in DR from four distinct perspectives (Figure 1). Initially, we provide an overview of the foundational technologies underpinning single-cell sequencing, focusing on mRNA-genome sequencing, isolation techniques, specific protocols, and the analysis of scRNA-seq data. Next, we examine DR research through the lens of scRNA-seq, juxtaposing its findings with those from complementary multi-omics approaches, including transcriptomics, genomics, metabolomics, and lipidomics. We delineate key cell subpopulations and their dynamic changes throughout DR progression. The synergistic potential of integrating scRNA-seq with these multi-omics techniques for advancing DR research is also emphasized. Furthermore, we provide an overview of the current challenges and future opportunities in translating scRNA-seq findings into novel therapeutics for DR.

## 2 Technical approaches

### 2.1 Technical approaches

In recent years, scRNA-seq (Ding et al., 2020) has revolutionized our understanding of cellular heterogeneity and regulation within diverse tissues during development and disease. This technology enables high-throughput, high-resolution transcriptomic profiling of individual cells by isolating and sequencing their RNA, thereby revealing distinct cellular states and functions (Lei et al., 2021).

#### 2.1.1 ScRNA-seq workflow

**FIGURE 2:**
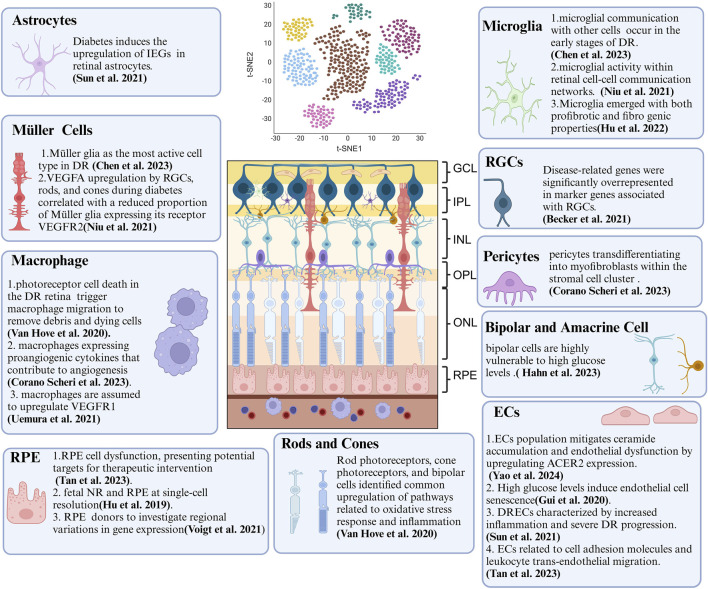
Summary of Crucial Cellular Components and Their Features in DR Revealed by scRNA-seq. ScRNA-seq technology provides biological information at the single-cell level and has been extensively applied to DR research. This review concludes that DR-associated microenvironments are characterized by diverse, highly heterogeneous, and dynamic cell populations. scRNA-seq has been instrumental in identifying various temporal states of different cell types, each with a distinct transcriptional signature and function. As scRNA-seq technology matures, it offers new approaches to study DR, deepening our understanding of its pathogenesis and paving the way for innovative treatments. Key cellular components include endothelial cells (ECs), retinal pigment epithelium (RPE), and retinal ganglion cells (RGCs).

The scRNA-seq workflow begins with the collection (Liu et al., 2024) and isolation of single cells from DR tissues (Chen et al., 2024b). Unlike conventional methods, scRNA-seq enables the differentiation of various retinal cell types, including ganglion cells, cone photoreceptors, and rod photoreceptors, at the single-cell level (Zhang et al., 2024). Figure 2 illustrates the essential steps involved in a typical scRNA-seq experiment.

### 2.2 Single-cell isolation

In DR, single-cell isolation techniques are essential due to the retina’s intricate tissue architecture (Niu et al., 2021), the need to identify rare cell populations, and the complexity of the generated data (Xu et al., 2021). These techniques (Kleino et al., 2022), coupled with high-throughput sequencing and bioinformatics, provide profound insights into DR pathogenesis and potential therapeutic targets (Van de Sande et al., 2023). Table 1 summarizes these experimental techniques, outlining their respective advantages and disadvantages.

**TABLE 1:** The specific nuances of each single-cell isolation method.

Techniques method	Throughput	Precision	Specificity	Advantages	Disadvantages	Applications	References
Micromanipulation	Low	High	High	minimal contamination	Labor-intensive; time-consuming	Rare cell isolation, research labs	Xu et al. (2023a)
Laser Capture Microdissection	Low	High	High	can isolate cells from complex tissues	potential thermal damage	Tissue-specific cell isolation	Xu et al. (2023b)
Optical Tweezers	Low	Very High	High	non-invasive; no need for labels	expensive; potential photodamage	Biophysics, cell manipulation	Deng et al. (2024)
Magnetic-Activated Cell Sorting	Medium	Medium	Medium	Gentle on cells; quick and easy	Lower purity; limited to surface markers	Cell separation, clinical labs	Tan et al. (2023)
Dielectrophoretic	Medium	Medium	Low	Label-free; gentle on cells; can sort based on intrinsic properties	complex instrumentation; potential cell viability issues	Label-free cell sorting	Sun et al. (2024)
Fluorescence-Activated Cell Sorting	High	High	High	High throughput; high purity; can isolate live cells	Expensive; requires fluorescent labeling; potential cell damage	Immunology, cancer research, sorting	Gérard et al. (2020)
Microfluidics	High	High	High	minimal reagent use; integrates with downstream analysis	Complex fabrication and operation; expensive setup	Single-cell sequencing, research	Wang et al. (2024)
Droplet-Based Microfluidics	Very High	Medium	High	reduces cross-contamination	Requires specialized equipment; droplet stability issues	Single-cell RNA sequencing, genomics	Wang et al. (2024)

### 2.3 Specific protocols for scRNA-seq

Specific protocols for scRNA-seq in DR research necessitate meticulous tissue preparation to ensure high cell viability (Heumos et al., 2023). This involves marker-based cell sorting to identify and isolate rare cell populations. Subsequent data integration with other omics approaches and advanced bioinformatics analysis are crucial for unraveling disease mechanisms and identifying potential therapeutic targets (Gérard et al., 2020). This methodology empowers comprehensive transcriptomic analysis at the single-cell level (Temple, 2023).

### 2.4 Analysis of single-cell sequencing data

ScRNA-seq is revolutionizing our comprehension of DR by facilitating the identification of differentially expressed genes (DEGs) and signature gene profiles (Wang et al., 2022). This technology affords granular insights into the cellular and molecular underpinnings of DR through comparative analysis of gene expression between healthy and diseased retinal cells (Zhang X et al., 2023). The retina’s diverse cellular composition, including ganglion, cone, and rod cells, necessitates specialized algorithms for precise classification and analysis (De Rop et al., 2022). DR-specific pathological alterations in retinal cells demand tailored analytical approaches to detect and interpret these changes at the single-cell level (Yu et al., 2022). To accurately capture the progressive nature of DR, single-cell data analysis must track evolving cellular states and interactions over time (Replogle et al., 2020). Understanding the intricate interplay between various retinal cell types within the microenvironment is pivotal for elucidating DR pathogenesis (Vu et al., 2016). Advanced computational methodologies, such as machine learning, enhance DEGs detection and provide deeper insights into disease mechanisms (McNulty et al., 2023). Integration with other omics data, enabled by tools like Cell Ranger.

## 3 ScRNA-seq application in diabetic retinopathy research

The advent of novel sequencing technologies has been instrumental in overcoming the limitations of traditional approaches and advancing our comprehension of DR pathogenesis. Advancements in scRNA-seq have provided invaluable insights into the heterogeneity of cell types and their corresponding states (Zhang P et al., 2023). In 2020, Van Hove et al. (2020) pioneered a single-cell transcriptomic retinal atlas, correlating genes associated with DR risk with cell type-specific expression patterns. Their findings emphasized distinct fibrotic, inflammatory, and gliotic profiles within macroglia subpopulations. Recent investigations have identified a unique retinal EC population in diabetes alongside a negative feedback regulatory pathway that attenuates endothelial dysfunction by upregulating alkaline ceramidase 2 (ACER2) expression to decrease ceramide content (Yao et al., 2024).

**FIGURE 3:**
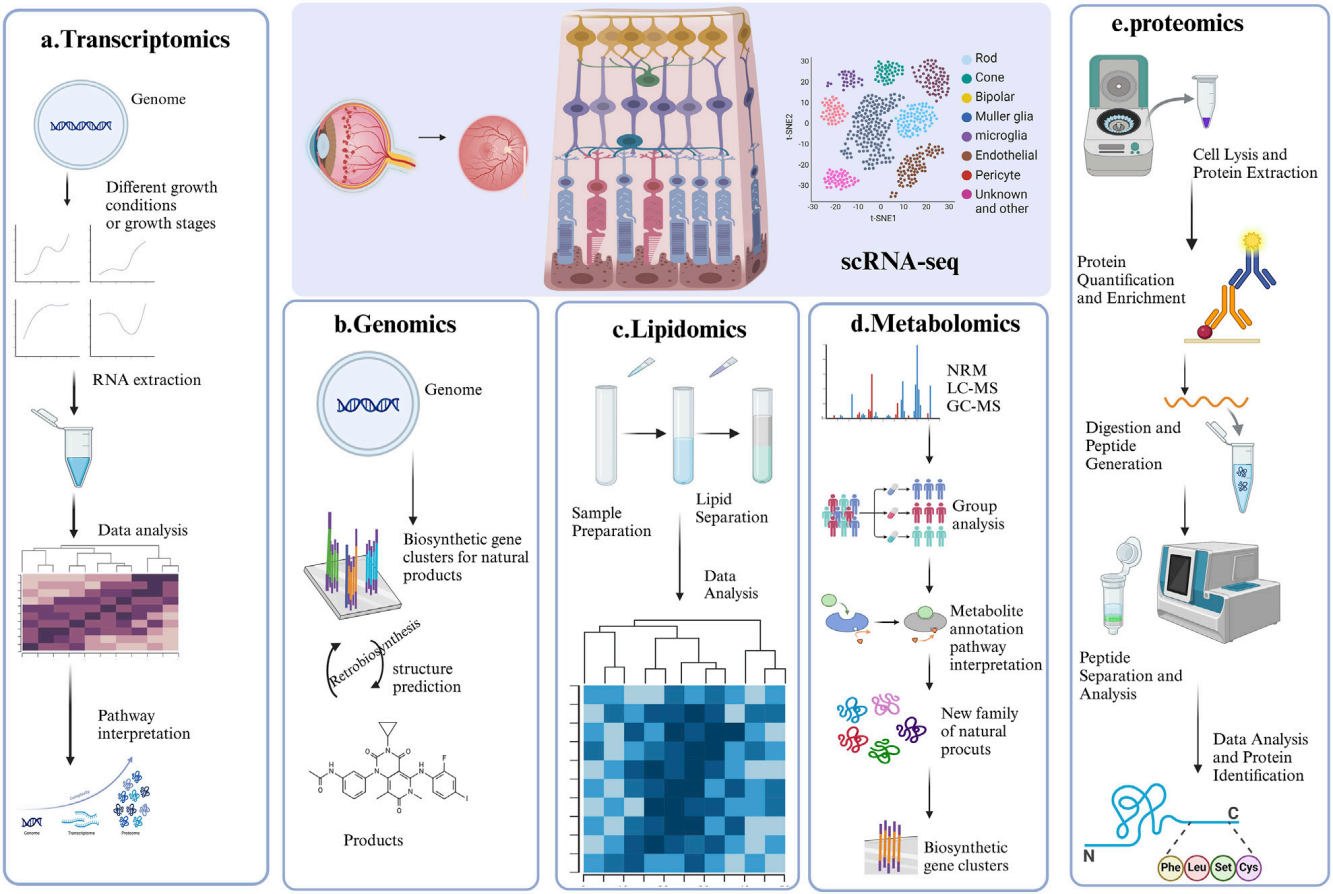
Representative strategy of single-cell RNA sequencing (scRNA-seq) and Multi-Omics. This figure summarizes the four key applications of single-cell RNA sequencing in DR research, each accompanied by a corresponding graph for better understanding. **(A)** ScRNA-seq combined with Transcriptomics; **(B)** ScRNA-seq combined with Genomics; **(C)** ScRNA-seq combined with lipidomic; **(D)** ScRNA-seq combined with Metabolomic; **(E)** ScRNA-seq combined with proteomics.

ScRNA-seq provides a highly sensitive method to detect gene expression changes within specific cell types, significantly enhancing our understanding of the molecular pathways involved in DR (Jovic et al., 2022). Identification of DEGs sheds light on the cellular and molecular mechanisms contributing to DR, thus facilitating the development of targeted therapies (Bawa et al., 2022). These gene expression alterations lead to dysfunction and subsequent injury across various retinal cell types, including retinal pigment epithelium (RPE), cone photoreceptors, macroglia, microglia, and vascular cells (Ghita et al., 2023). Furthermore, genetic risk variants, cellular statuses, and microenvironmental deterioration further propel DR progression. This section will explore the diverse applications of scRNA-seq in DR research (Figure 3; Table 2 for details).

**TABLE 2:** Various applications of ScRNA-seq in DR research (n = 26 studies).

Technical approach	Study references	Type of cells	Modeled DR feature	Analysis	Species	Sample and model	Number of cells	Key study findings
ScRNA-seq	Xia et al. (2023)	Photoreceptors, ECs, amacrine, BCs, pericytes, pigmented epithelial cells, microglial, Müller, erythroid cells, ganglion cells	Vascular diseases	10×Genomics	Mice	Control (n = 2) OIR (n = 2)	76,000	Identified retinal pericytes sub-population 2, marked by Col1a1 was vulnerable to retinal capillary dysfunction
ScRNA-seq Transcriptomic	Van Hove et al. (2020)	Photoreceptors, BCs, amacrine cells, macroglia, immune cells, ECs	Degenerating diabetic retina	10×Genomics	Mice	Akimba (n = 4) Control (*n* = 2)	9,474	Identified in DR are revealing unique functional subtypes of inflammatory and macroglia cells
ScRNA-seq Transcriptomic	Corano Scheri et al. (2023)	ECs, pericytes, glia, neurons, immune cells	Preretinal fibrovascular membranes	10×Genomics	Human	Control (n = 4) PDR (n = 4)	4,097	Identified is AEBP1 signaling modulates the transformation of pericytes into myofibroblasts
ScRNA-seq Genomics	Niu et al. (2021)	Rods, cone, BCs, Müller glia, amacrine, microglia, ganglion cells, cones, pericytes, horizontal cells	DR-associated neurovascular degeneration	10×Genomics	Mice	Control (n = 3) DR (n = 3)	51,558	Identified that the expression of RLBP1 is reduced in diabetes, and Müller glia mitigates neurovascular degeneration associated with DR
ScRNA-seq Genomics	Wang J et al. (2024)	Rods, cone, BCs, Müller glia, amacrine, microglia, ganglion cells, cones, pericytes, horizontal cells	DR-associated neurovascular degeneration	10×Genomics	Mice	Control (n = 3) DR (n = 3)	51,558	Identified that the expression of RLBP1 is reduced in diabetes, and Müller glia mitigates neurovascular degeneration
ScRNA-seq Transcriptomic	Wang J et al. (2024)	BCs, amacrine, ganglion cells, cones, microglia, ECs, pericytes	Biomarkers and therapeutic targets	10×Genomics	Human	PDR (n = 5) Control (n = 3)	5,011	Identified are key meta-programs elucidating the role of microglia in the pathogenesis of PDR, along with three critical ORGs
ScRNA-seq Transcriptomic	Becker et al. (2021)	Amacrine, BCs, ECs, horizontal cells, RGC, Rod, microglia, Müller	Neurological damage	Seurat R package72	Human	Human (n = 80)	3,223	Identified is the continuous downregulation of RGC-specific genes in DR
ScRNA-seq Genomics Transcriptomic	Chen et al. (2023a)	Rod, cone, Müller, horizontal cells, BCs, amacrine, ganglion, microglia, ECs, pericytes, astrocyte, T cells	Neural retinas damage	10×Genomics	Human Mice	BKSDB(n = 2) BKSWT (n = 2)	276,402	Identified the expression of RLBP1 is reduced in diabetes, and Müller glia mitigates neurovascular degeneration associated with DR
ScRNA-seq	Sun et al. (2021)	photoreceptor, BCs, amacrine cells, horizontal cells, ganglion cells	vascular inflammation	10×Genomics	Mice	Control (n = 3) Diabetic (n = 3)	14,000	Identified the expression of RLBP1 is reduced in diabetes, and Müller glia mitigates neurovascular degeneration associated with DR
ScRNA-seq Transcriptomic	Saddala et al. (2023)	Microglia, Rods, cone, BCs, Müller glia, amacrine cells, RGCs, cones	retinal degeneration	10×Genomics	Mice	Control (n = 5) DR (n = 8)	6,800	Identified is the analysis of retinal homeostasis and microglial degeneration
ScRNA-seq Transcriptomic	Wang J et al. (2024)	BCs, amacrine, ganglion cells, cones, microglia, ECs, pericytes	Biomarkers and therapeutic targets	10×Genomics	Human	PDR (n = 5) Control (n = 3)	5,011	Identified are key meta-programs elucidating the role of microglia in the pathogenesis of PDR, along with three critical ORGs
ScRNA-seq Transcriptomic	Becker et al. (2021)	Amacrine,BCs,ECs, horizontal cells, RGC, Rod, microglia,Müller	Neurological damage	Seurat R package72	Human	Human (n = 80)	3,223	Identified is the continuous downregulation of RGC-specific genes in DR
ScRNA-seq Genomics Transcriptomic	Chen et al. (2023b)	Rod,cone,Müller,horizontal cells, BCs, amacrine, ganglion, microglia, ECs, pericytes, astrocyte, T cells	Neural retinas damage	10×Genomics	Human Mice	Human (n = 4) BKSDB(n = 2) BKSWT (n = 2)	276,402	Identified the expression of RLBP1 is reduced in diabetes, and Müller glia mitigates neurovascular degeneration associated with DR
ScRNA-seq	Sun et al. (2021)	photoreceptor, BCs, amacrine cells, horizontal cells, ganglion cells	vascular inflammation	10×Genomics	Mice	Control (n = 3) Diabetic (n = 3)	14,000	Identified the expression of RLBP1 is reduced in diabetes, and Müller glia mitigates neurovascular degeneration associated with DR
ScRNA-seq Transcriptomic	Saddala et al. (2023)	Microglia, Rods, cone, BCs, Müller glia, amacrine cells, RGCs, cones	retinal degeneration	10×Genomics	Mice	Control (n = 5) DR (n = 8)	6,800	Identified is the analysis of retinal homeostasis and microglial degeneration
ScRNA-seq Transcriptomic	He et al. (2024)	ECs, rod, cone, Müller, horizontal cells, BCs, amacrine cells, ganglion cells, pigment epithelium cells, microglia, pericytes, astrocyte	retinal vasculature	10×Genomics	Mice Human	Mice (n = 3) Human (n = 54)	18,439	Identified is that GLP-1 expression is downregulated in DR, whereas GLP-1 RAs increase GLP-1R expression and improve retinal degeneration
ScRNA-seq Metabolomic	Yao et al. (2024)	microvascular ECs, microglia, pericytes, photoreceptors, Müller, neurons	Blood-Retina- Barrier (BRB)	10×Genomics	Mice	Diabetic (n = 2) Control (n = 2)	18,376	Identified a new type of EC specific to diabetes and a regulatory pathway
ScRNA-seq Transcriptomic	Zhou et al. (2024)	B cell, monocyte, EC, Osteoblasts, macrophage, T cell	immune cell infiltration	10×Genomics	Human	Control (n = 3) FVM (n = 3)	6,894	Identified the location of HMOX1 expression, TP53, HMOX1, and PPARA related to M2 macrophages and ferroptosis
ScRNA-seq Transcriptomic	Xiao et al. (2021)	Microglia, Müller, photoreceptors photoreceptors, amacrine, BC	retina neurodegeneration	10×Genomics	Monkey	Control (n = 2) diabetes (n = 2)	10,263	Identified cell-type-specific molecular changes and constructing the retinal interactome
ScRNA-seq Genomics	Xu et al. (2023a)	microglia, fibroblasts, EC, dendritic cells, pericytes	vasculature of DR and AD	10×Genomics	Human	PDR (n = 2), AD (n = 9)	102,451	Identified APP signaling is crucial in the vasculature of both PDR and AD
ScRNA-seq Proteomics	Wang J et al. (2024)	microglia, lymphocytes, myeloid cells, endothelial	PDR	10×Genomics	Human	Control (n = 3) PDR (n = 5)	5,000	Identified key cellular mechanisms, oxidative stress-related genes, and potential therapeutic targets for proliferative diabetic retinopathy
ScRNA-seq Transcriptomic	Chen et al. (2023b)	photoreceptors, cone BCs, Müller cells, horizontal cells, epithelial, ECs	aging process	10×Genomics	Mice	DR (n = 3) Control (n = 3)	51,327	Identified p53 accelerates EC senescence and exacerbates the progression of diabetic retinopathy
ScRNA-seq Transcriptomic	Mao et al. (2023)	rod, cone, bipolar cell, Müller glia, amacrine cell, horizontal cell, EC, RGC,microglia, pericyte	apoptosis	10×Genomics	Mice	DR (n = 3) Control (n = 3)	51,558	Identified is the first single-cell atlas of alternative transcription start sites in healthy and diabetic retinas
ScRNA-seqTranscriptomic	Gao et al. (2022)	Macrophages, Monocytes, B cells, T cells, Fibroblasts	Proliferative Diabetic Retinopathy	10×Genomics	Human	PDR (n = 11) Control (n = 7)	7,971	Identified CD44, ICAM1, and POSTN were associated with PDR, suggesting POSTN as a key ligand
ScRNA-seq Transcriptomic	Zhang J et al. (2022)	Retinal pigment, ECs, astrocytes, microglia, anaplastic cells, cone, rod, BCs, Müller, ECs, T cells	oxidative stress- inflammation	10×Genomics	Mice	Healthy (n = 1) DR (n = 1)	21,588	Identified in DR tissues, Müller glia, microglia, ECs, and BCs are involved in pathways related to oxidative stress and inflammation
ScRNA-seq Transcriptomic	Liao et al. (2024)	NK cells, monocytes, B cells, dendritic cells, basophils plasmacytoid dendritic cells	circulating immune	10×Genomics	Human	Control (n = 5) NDR (n = 3) DR (n = 3)	71,545	Identified a detailed map of immune cells in patients with type 1 DR, revealing that the JUND gene plays an important role in its development
ScRNA-seq Transcriptomic	Deng et al. (2024)	rod, cone, amacrine cells, bipolar cell, Müller cells, microglia, ECs, horizontal cells, macrophages, pericytes	neural retinas damage	10×Chromium	Rat Human	Control (n = 2)DR (n = 3) Macula (n = 3) Peripheral (n = 3)	23,724	Identified Müller cell clusters upregulate the Rho gene in response to damaged photoreceptors across species, with co-expression of RHO and PDE6G
ScRNA-seq Transcriptomic Metabolomics Lipidomics	Lv K et al. (2022)	microglia, rods, monocytes, cones, BCs, amacrine cells, horizontal cells, RGCs	Inflammation	10×Chromium	Mice	Control (n = 3) DR (n = 3)	14,355	Identified activated microglia in the retina may change their internal metabolism, which can lead to inflammation in the early stages of DR
ScRNA-seq Transcriptomic	Wang Y et al. (2022)	rod, cone, horizontal cells, amacrine cells, BCs, Müller cells, microglia cells, ECs, pericytes	BRB	10×Chromium	Rat	Control (n = 2), Diabetic (n = 3)	35,910	Identified is an atlas of the inner BRB for the early stage of DR, elucidating the degeneration of its constituent cells and Müller cells
ScRNA-seq Transcriptomic	Wang Y et al. (2024)	rod, cone, Müller, horizontal, amacrine, bipolar, ECs, pericytes, microglia	Inflammation	10×Chromium	Rat	Control (n = 2), Diabetic (n = 3)	35,910	Identified are the most pronounced differential expression changes in microglia in early DR
ScRNA-seq Transcriptomic	Xu et al. (2023a)	mesangial cell, cone, podocyte, intercalated cell, principal cell, proximal tubule cell, rod, ganglion cell, bipolar cell, horizontal, T cell myeloid cell	Molecular of DN and DR	10×Chromium	Mice	Control (n = 3), Diabetic (n = 8)	9,264	Identified that the association between MCs and RPCs is a cellular basis for the cooccurrence of DN and DR

ScRNA-seq, single-cell RNA, sequencing; DR, diabetic retinopathy; GWAS, Genome-Wide Association Studies; RPE, retinal pigment epithelium.

### 3.1 ECs

ECs are critical for the formation and maintenance of the blood-retinal barrier and retinal vascular integrity (Yang and Liu, 2022). In DR, EC dysfunction underlies vascular leakage and neovascularization. ScRNA-seq has become a valuable tool for identifying gene expression alterations in ECs that contribute to these vascular pathologies, providing novel insights into the mechanisms of vascular permeability and abnormal blood vessel growth (Hu et al., 2023).

Studies employing scRNA-seq have generated comprehensive transcriptional profiles of retinal cells in DR. Yao et al. (2024) characterized the heterogeneity and functional diversity of retinal ECs in the human choroid, identifying a novel diabetes-specific EC population. They further elucidated a negative feedback regulatory pathway that attenuates ceramide accumulation and endothelial dysfunction by upregulating ACER2 expression. These findings emphasize the crucial interplay between ECs and other cellular components in DR. Subsequent research by Gui et al. (2020) and Bertelli et al. (2022) reinforced the pivotal role of ECs in DR progression. High glucose levels were found to induce EC senescence, with p53 identified as a key regulator of this process. Inhibition of p53 in human retinal microvascular ECs diminished senescence markers, while overexpression exacerbated them. Sun et al. (2021) constructed a transcriptome atlas encompassing over 14,000 single cells from both healthy and diabetic murine retinas. This analysis identified a distinct subgroup of ECs, termed diabetic retina-specific ECs, characterized by heightened inflammation and accelerated DR progression. The study linked the HIF-1 signaling pathway to dysregulated genes in both general diabetic ECs and DRECs, emphasizing its pivotal role in DR pathogenesis. Subsequent analysis of 80 human post-mortem retinal samples from patients with varying DR stages demonstrated significant enrichment of DR-associated genes in ECs, specifically those linked to cell adhesion molecules and leukocyte trans-endothelial migration (Tan Y et al., 2023).

The application of scRNA-seq has markedly propelled DR research, enabling the identification of ECs subclusters exhibiting noncanonical transcriptional profiles and active angiogenesis. This high-resolution methodology provides unparalleled insights into the cellular and molecular underpinnings of DR, thereby facilitating the development of innovative therapeutic approaches.

### 3.2 RPE

RPE cells are crucial for maintaining photoreceptor cell function and overall retinal health (Liu et al., 2022a). Dysfunction of RPE cells in DR can lead to neural retinal (NR) degeneration and subsequent visual impairment. ScRNA-seq has emerged as a powerful tool for identifying distinct RPE cell populations based on their unique gene expression profiles, providing invaluable insights into the molecular mechanisms underlying RPE cell dysfunction in diabetic conditions.

Over the years, studies utilizing scRNA-seq have identified substantial alterations in gene expression associated with oxidative stress, inflammation, and cellular metabolism within RPE cells during DR (Tan Y et al., 2023). These investigations have pinpointed key regulatory pathways and genes implicated in RPE cell dysfunction, providing potential targets for therapeutic interventions. Notably, Voigt et al. (2021a) isolated RPE tissue from the macula and periphery of three human donors to examine regional variations in gene expression. Their research culminated in a comprehensive expression atlas of RPE and choroidal cell types, significantly surpassing the resolution of previous bulk transcriptomic studies of the RPE. Another study (Hu et al., 2019) characterized the transcriptome of human fetal NR and RPE at single-cell resolution, revealing distinct gene expression profiles: NR genes linked to nervous system development and RPE genes associated with retinol metabolism. Despite these differences, both tissues exhibited developmental processes ranging from active proliferation to visual perception maturation, suggesting functional interactions during later developmental stages. Disruptions in these interactions have been implicated in the formation of a multilayered retina-like structure by the RPE.

The application of scRNA-seq to individual RPE cells has significantly advanced our comprehension of normal visual physiology and the dysfunctions associated with DR. By providing granular insights into the gene expression profiles and regulatory pathways of RPE cells, scRNA-seq research holds the potential to inform novel therapeutic strategies targeting RPE dysfunction and its contribution to DR.

### 3.3 Retinal ganglion cells (RGCs)

RGCs are essential for relaying complex, integrated visual information from the retina to the brain’s visual centers (Kim et al., 2021). In DR, damage to RGCs is a primary cause of vision loss (Zhang et al., 2017). ScRNA-seq has provided compelling evidence of RGC depletion in the early stages of DR.

A comprehensive study analyzed 80 human post-mortem retinal samples from 43 patients with varying stages of DR using RNA sequencing (Becker et al., 2021). This investigation identified disease-related genes that were significantly overrepresented among marker genes associated with RGCs, indicating a progressive downregulation of RGC-specific genes during DR progression and suggesting a general loss of this cell type. Notably, the study revealed partial RGC loss even in diabetic patients without clinically diagnosed DR, aligning with optical coherence tomography findings of RGC damage in this pre-pathological stage of DR. These results underscore the critical importance of early detection and intervention to prevent further RGC loss and preserve vision in diabetic patients (Lee et al., 2021). One study (Yang Z et al., 2023) investigates Using scRNA-seq, EMPA mitigates microglia-mediated neuroinflammation and prevents RGC loss in retinal injury, with Mfn1 and Opa1 as essential contributors to its mitochondrial protective effects.

Notably, RGCs show substantial diversity, with each subtype expressing different members of the synuclein family. ScRNA-seq has revealed cross-species expression patterns of synuclein family members, offering new avenues to investigate the distinct roles of RGC subtypes in visual signal processing and transmission (Laboissonniere et al., 2019; Peng et al., 2024). These findings hold promise for clinical advancements, potentially leading to novel diagnostic and therapeutic approaches for retinal and neurodegenerative diseases.

### 3.4 Müller cells

Müller cells are essential for providing structural and functional support to retinal neurons (Tang et al., 2023). In the context of DR, these cells undergo reactive gliosis, compromising their ability to maintain retinal homeostasis (Perais et al., 2023). scRNA-seq has been instrumental in elucidating the molecular alterations within Müller cells during DR, particularly in genes associated with inflammation, gliosis, and their supportive roles in the retina.

Employing scRNA-seq, Chen et al. (2023b) identified Müller glia as the most active cell type under these conditions. While overall communication among Müller glia remained consistent, a decrease in interactions mediated by growth factors and a corresponding increase in cytokine-driven interactions were observed. In another study (Niu et al., 2021) utilizing scRNA-seq, Müller glia cluster six was highlighted as exhibiting a stronger correlation with retinal alterations in diabetic mice compared to other clusters.

Moreover, diabetic conditions were associated with upregulated VEGFA expression in RGCs, rods, and cones. Concurrently, there was a decreased proportion of Müller glia expressing its receptor, VEGF receptor 2(VEGFR2). These findings highlight the complex molecular adaptations occurring within Müller cells in response to the diabetic environment. ScRNA-seq has provided invaluable insights into the multifaceted roles of Müller cells in DR, elucidating alterations in their gene expression profiles and interactions with other retinal cells in response to diabetes. These findings have identified potential therapeutic targets for modulating Müller cell function and mitigating retinal damage associated with DR.

### 3.5 Photoreceptors cells (rods and cones)

Photoreceptor cells are essential for converting light into neural signals. Their degeneration, significantly contributing to vision loss in the advanced stages of DR, has been a focal point of research (Rauscher et al., 2024). ScRNA-seq has been instrumental in elucidating alterations in gene expression within photoreceptors under hyperglycemic and ischemic conditions, providing crucial insights into the molecular mechanisms underlying their degeneration (Xiong et al., 2019).

A recent study (Van Hove et al., 2020) reported substantial alterations in retinal cell population composition, notably impacting rod photoreceptors and specific inflammatory cell subsets. Differential gene expression analysis of rods, cones, and bipolar cells (BCs) identified a common upregulation of pathways associated with oxidative stress response and inflammation in Akimba models.

Through scRNA-seq, researchers have acquired a comprehensive understanding of the transcriptional heterogeneity within neuronal and inflammatory cell populations in DR. This in-depth knowledge offers significant potential for developing targeted therapies designed to mitigate photoreceptor degeneration and preserve vision in patients with DR.

### 3.6 Microglia

Microglia, the resident macrophages of the retina, play crucial roles in both inflammation and neuroprotection within the tissue (Kinuthia et al., 2020). In DR, activated microglia can contribute to inflammatory damage. ScRNA-seq has been instrumental in characterizing the diverse activation states of microglia and macrophages, distinguishing between pro-inflammatory and neuroprotective phenotypes, and identifying the underlying signaling pathways involved in their activation (Van Hove et al., 2020).

A study (Chen et al., 2023b) observed microglial activation in BKSDB mice, suggesting impaired communication with other cell types during the early stages of DR. Analysis revealed disparate changes in receptor-ligand interactions, with decreased microglial communication mediated by growth factors and enhanced interactions *via* cytokines and other signaling pathways in BKSDB compared to BKSWT mice. Another study (Niu et al., 2021) reported elevated microglial activity within retinal cell-cell communication networks in BKSDB mice at 8 months of age. Wang Y et al. (2024) reveals significant early-stage changes in retinal microglia under diabetic conditions, identifying three new microglial subtypes and constructing an inflammatory network, which provides insights into early intervention strategies for DR through scRNA-seq analysis.

ScRNA-seq of fibrovascular membranes in a study of PDR identified eight distinct cellular populations, with microglia being the predominant cell type (Hu et al., 2022). A subpopulation of microglia expressing Glycoprotein Non-Metastatic Protein B exhibited both profibrotic and fibrogenic characteristics, suggesting a potential role in PDR progression. Pseudotime analysis revealed unique differentiation trajectories of profibrotic microglia from resident microglia within the PDR context. The identification of ligand-receptor interactions between profibrotic microglia and upregulated cytokines in PDR vitreous implied their activation within the PDR microenvironment.

The above studies overlap in their assertion of distinct microglial phenotypes in DR, providing novel insights into the cellular and molecular underpinnings of DR pathogenesis.

### 3.7 Astrocytes

Astrocytes, glial cells that support neuronal function and maintain the integrity of the blood-retinal barrier, are essential for retinal homeostasis. These cells exhibit a dynamic response to injury and inflammation (Xia et al., 2022). ScRNA-seq enables precise characterization of astrocytes, differentiating them from other glial cell types based on specific marker genes (Shi et al., 2023). This technique has elucidated molecular alterations in astrocytes under diabetic conditions, including changes in gene expression related to neuroinflammation and gliosis (Pun et al., 2023). Through scRNA-seq, researchers have investigated the role of astrocytes in DR, focusing on their involvement in inflammation and neurovascular damage. Diabetes has been shown to induce the upregulation of immediate early genes in retinal astrocytes (Shi et al., 2023). By identifying a subset of ECs exhibiting heightened deregulation, specifically in diabetic retinas, termed DRECs, researchers have elucidated pathways associated with these genes. These findings underscore the substantial contribution of astrocytes to DR progression (Pun et al., 2023).

### 3.8 Macrophage

Macrophages, differentiated mononuclear phagocytes, are essential for inflammation, tissue repair, and immune responses. These cells phagocytose pathogens, cellular debris, and apoptotic cells, and secrete cytokines to regulate immune function (Wu et al., 2021). In DR, macrophages contribute to neuroinflammation and tissue damage by releasing pro-inflammatory cytokines and mediating immune responses. ScRNA-seq identifies macrophages based on their expression of marker genes such as CD68 and CD163, as well as various cytokines, revealing gene expression profiles associated with phagocytosis, antigen presentation, and inflammation (Cochain et al., 2018).

Van Hove et al. (2020) identified significant enrichment of pathways associated with the activation of inflammatory monocyte-derived macrophages, microglia, macrophages, leukocytes, and lymphocytes, as well as monocyte and leukocyte differentiation. These findings suggest a potential contribution to DR progression. Conversely, extensive photoreceptor cell death in the diabetic retina may induce macrophage migration to clear cellular debris. Further analysis of inflammatory cells revealed a macrophage subcluster expressing proangiogenic cytokines, implicating these cells in angiogenesis (Corano Scheri et al., 2023). Monocyte-derived macrophages are implicated in upregulating VEGF receptor 1 (VEGFR1) (Uemura et al., 2021) in various tissues, including the retina. Activation of VEGFR1 in these mononuclear phagocytes enhances their production of pro-inflammatory and pro-angiogenic cytokines such as CC chemokine ligand 2, interleukin-1β, interleukin-6, tumor necrosis factor-α, and VEGFA (Van Bergen et al., 2019). Based on these findings, targeting ischemia-related M1-like macrophages may represent a novel therapeutic approach for DR.

### 3.9 Pericytes

While pericyte degeneration is a feature of other diseases, its severity in the retina during DR suggests the involvement of unique intraocular microenvironmental factors in pericyte loss (Spencer et al., 2020). ScRNA-seq profiling of pericytes has the potential to illuminate pathways essential for their survival and function, thereby identifying therapeutic targets to preserve retinal vascular integrity (Zhang et al., 2022). Studies of DR patients have identified a subpopulation of pericytes undergoing transdifferentiation into myofibroblasts within the stromal cell compartment (Corano Scheri et al., 2023). Adipocyte Enhancer-binding Protein 1 (AEBP1) exhibits significant upregulation in these myofibroblast clusters, suggesting its involvement in this transformation process. Experiments exposing human retinal pericytes to high-glucose conditions have confirmed this molecular shift, with siRNA-mediated knockdown of AEBP1 attenuating the expression of profibrotic markers. These findings provide critical insights into the molecular mechanisms underlying pericyte dysfunction in DR.

### 3.10 Bipolar and amacrine cells

Bipolar and amacrine cells integrate signals from photoreceptors and relay them to RGCs (Soni et al., 2021). Studies (Shekhar et al., 2016; Matosin et al., 2023) indicate that BCs are particularly susceptible to hyperglycemia. While diabetic mouse retinas exhibit BC-intrinsic defense mechanisms against diabetes-induced degeneration, a recent study Chen et al. (2023b) employed massively parallel scRNA-seq and computational analysis to categorize approximately 25,000 mouse retinal BCs into 15 subtypes. These included previously characterized subtypes and two novel subtypes, one displaying atypical morphology and localization (Ising et al., 2019).

ScRNA-seq is a systematic approach enabling comprehensive molecular classification of neurons, identification of novel neuronal subtypes, and elucidation of intra-class transcriptional heterogeneity. This technique is instrumental in advancing our comprehension of the cellular and molecular mechanisms underlying DR, particularly in understudied cell types such as BCs.

## 4 Application of scRNA-seq with multi-omics for DR research

**FIGURE 4:**
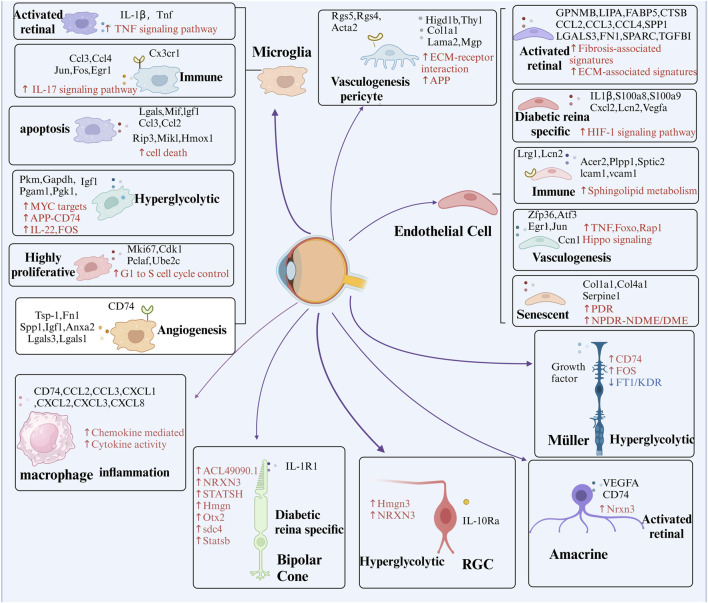
Presents the identification of key cell subpopulations in DR through multi-omics analysis. The figure highlights distinct cell subpopulations to emphasize the cellular heterogeneity observed during DR progression, offering insights into the specific roles of different cell clusters in the disease’s pathophysiology. Red arrows and text represent pathways that are significantly upregulated within these subpopulations, while blue arrows and text signify pathways that are downregulated. Additionally, black text denotes unique intracellular or secreted proteins, as well as membrane receptors, that are specifically expressed within each subpopulation.

Omics analyses aim to characterize the diverse molecules essential to life, but individual omics datasets often fail to capture the complexity of molecular interactions. Multi-omics integrates data from genomics, transcriptomics, proteomics, metabolomics, lipidomics, epigenomics and spatial transcriptomics to provide a comprehensive perspective (Beneyto-Calabuig et al., 2023). This holistic approach facilitates the exploration of relationships across biological levels, deepening our understanding of molecular changes in development, cellular responses, and disease (Gan et al., 2024). Disruptions in cellular metabolic pathways, such as glucose, amino acid, and lipid metabolism, are also recognized as key pathogenic mechanisms in DR (Sun et al., 2021). Advances in technology, computational tools, and commercial platforms have significantly accelerated multi-omics analysis (Zhang Y et al., 2024). The emergence of single-cell omics extends this approach to the individual cell level, offering unprecedented insights into gene regulation (Sun et al., 2024). This integrated methodology has revealed intricate interactions between cell types and gene expression, providing novel perspectives on DR, as depicted in Figure 3. The identification of unique cell clusters and their transformations in DR facilitates a deeper understanding of the disease’s pathogenesis and potential therapeutic interventions (Figure 4).

### 4.1 ScRNA-seq combined with genomics

Advances in genetic research have elucidated the complex mechanisms underlying DR (Kang and Yang, 2020). Integrating scRNA-seq with genetic association studies offers a powerful approach to identifying susceptibility genes for DR (Corano Scheri et al., 2023). Traditionally, gene expression signatures have been derived from conventional molecular profiling methods, such as microarray and genomics, which assess average gene expression across entire cell populations. To correlate gene expression with genotype, researchers often integrate single-cell transcriptomes and single-cell genomes, as summarized in Table 3, which highlights their combined benefits. Primarily, this approach enables the delineation of cell lineage and evolution. By analyzing both cellular expression profiles and genetic variants, investigators can reconstruct lineage relationships between different cell types and trace the evolutionary paths of cell populations over time (Weber et al., 2021). Such knowledge is essential for comprehending the development of diseases characterized by cellular heterogeneity, including DR.

**TABLE 3:** The differences between ScRNA-seq and Traditional Bulk RNA Sequencing.

Aspect	ScRNA-seq	Traditional bulk RNA sequencing
Sample Preparation	Requires individual cell isolation, often *via* droplet-based methods or sorting	Uses homogenized cell mixtures
Library Preparation	Involves reverse transcription of RNA from individual cells with UMIs and cell barcodes	Involves reverse transcription of RNA from pooled cells without barcodes
Sequencing	Captures transcriptome of each cell with high sequencing depth	Captures average gene expression of the population with lower sequencing depth
Data Analysis	Analyzes gene expression at the single-cell level, involving clustering and trajectory inference	Analyzes average gene expression across the population, focusing on differential expression between conditions
Resolution and Sensitivity	High resolution, reveals cell heterogeneity, identifies rare cell types; complex and costly	Simpler and less expensive, robust average expression profiles; masks cellular heterogeneity
Data Interpretation	Studies cell differentiation and variability with potential data noise and technical dropouts	Cleaner data with less noise, but unable to resolve individual cell differences
Applications	Ideal for developmental biology, cancer, immunology, and understanding cellular heterogeneity	Suitable for identifying overall gene expression changes between conditions

Furthermore, this integrated analysis facilitates the identification and characterization of cellular subpopulations. Researchers can identify and characterize specific subgroups defined by unique combinations of gene expression profiles and genetic mutations (Valecha and Posada, 2022). This capability is invaluable for studying the cellular diversity within complex tissues and identifying novel targets for therapeutic intervention. In contrast, scRNA-seq offers detailed profiling at the individual cell level, unveiling gene expression differences within distinct cell types. Consequently, scRNA-seq facilitates the identification of novel biomarkers associated with DR progression and response to targeted therapies.

One study (Zhang et al., 2019) presents the first unbiased and comprehensive cellular and transcriptional characterization of PDR retinal neovascular (RNV) membranes obtained during vitreoretinal surgery. They findings underscore the pivotal role of M2 macrophages in RNV formation and highlight the significant contribution of hyalocytes to the myofibroblast population. Further investigation is anticipated to identify additional factors that can improve prognostic prediction and uncover novel therapeutic targets. The integration of scRNA-seq with multi-omics methodologies holds the potential to revolutionize DR treatment by providing a deeper understanding of the cellular and molecular mechanisms underlying the disease, thereby facilitating the development of precise and efficacious therapeutic interventions.

Population-based GWAS have consistently identified multiple genetic variants associated with DR (McIntosh et al., 2024). Similar to other complex diseases, such as age-related macular degeneration, many of these DR-associated variants are located within non-coding regions and exert crucial regulatory functions on gene expression (Herrera-Luis et al., 2022). Notably, single nucleotide polymorphisms identified through meta-GWAS of DR are enriched in DNase hypersensitivity sites, suggesting their potential to influence gene expression by altering chromatin states or transcription factor binding (Khan et al., 2020).

Research by Chen et al. (2023b) identified rod BCs, cone BCs, and amacrine cells as retinal neuronal cell types most strongly associated with DR risk. The gene Neurexin 3, a DR risk candidate, is primarily expressed in interneurons, suggesting their critical role in DR pathogenesis. Subsequent research by Niu et al. (2021) employed scRNA-seq to classify 11 retinal cell types and analyze the cell-type-specific expression of DR-associated loci. These studies unveiled diverse expression patterns, including elevated acid-sensing ion channel five in specific cell clusters and altered expression of genes such as Fos, madd, and pttg1 across multiple cell types. In diabetic mice, Retinaldehyde Binding Protein 1 (RLBP1), encoding Cellular Retinaldehyde-Binding Protein, exhibited decreased expression in Müller glia but increased expression in other retinal cells. Furthermore, Calcium Voltage-Gated Channel Subunit Alpha1 H, encoding a calcium channel subunit, was downregulated in cone BCs during DR.

Importantly, research on proliferative DR in mice revealed associations between amyloid-beta precursor protein (APP) gene expression and Alzheimer’s disease (AD) risk genes identified through GWAS (Xu et al., 2023). APP expression exhibited negative correlations with SCIMP, ABI3, ABCA7, and APOE, and positive correlations with MINDY2 and ADAMTS1, suggesting a potential link to AD risk.

These findings offer a comprehensive characterization of the retinal environment under both diabetic and normal conditions, identifying novel pathogenic factors that may serve as therapeutic targets for DR and related disorders.

### 4.2 ScRNA-seq combined with transcriptomics

Alternative transcription starts sites (TSS) generate diverse 5′-UTR isoforms, influencing mRNA stability and translation (Golubnitschaja et al., 2024). Techniques such as Cap Analysis of Gene Expression and TSS-seq measure TSS in bulk tissues but mask cellular diversity, whereas scRNA-seq can profile cell-type-specific changes (Stuart et al., 2019). scRNA-seq enables detailed gene expression profiling at the individual cell level, identifying ligand-receptor interactions and decoding intercellular communication networks (Zhu et al., 2023). scRNA-seq provides detailed gene expression profiles at the individual cell level, revealing cellular heterogeneity and rare cell types, although with greater complexity and cost (Liu et al., 2024). In contrast, traditional bulk RNA sequencing provides simpler, cost-effective, and robust average expression profiles yet cannot resolve to detect differences between individual cells (Tan Y et al., 2023). Table 4 provides a comparison of scRNA-seq and bulk RNA analysis.

**TABLE 4:** The impact of using single-cell transcriptomes and single-cell genomes on DR.

Advantage	Description	Study	Findings	Impact	References
Enhanced Resolution	Provides a detailed view of the relationship between genetic mutations and gene expression in individual cells	DR Research	Combined scRNA-seq and scDNA-seq revealed specific genetic mutations linked to altered gene expression profiles in retinal cells	Improved understanding of cellular changes in diabetic retinopathy progression	Niu et al. (2021)
Understanding Heterogeneity	Helps in identifying cellular heterogeneity within tissues, uncovering distinct cell subpopulations and their genetic makeup	Developmental Biology	Integration of scRNA-seq and scDNA-seq identified lineage-specific gene expression changes linked to developmental mutations	Insights into developmental pathways and genetic regulation	Deng et al. (2024)
Disease Mechanisms	Facilitates the study of how specific genetic alterations drive disease progression at the cellular level, particularly in complex diseases like DR	DR Research	Identified genetic mutations associated with increased vascular permeability and neovascularization	Improved understanding of disease mechanisms leading to new therapeutic targets	Cheng Y et al., (2024)
Drug Response	Aids in understanding differential drug responses based on genetic and transcriptional profiles, leading to more targeted therapies	Drug Response	Linked genetic variants to differential gene expression in response to DR treatments	Identification of biomarkers for predicting treatment efficacy and resistance	Abbas et al. (2024)
Developmental Biology	Offers insights into developmental processes by linking genetic changes to expression patterns during different stages of development	Developmental Biology	Tracked gene expression changes during development in conjunction with genetic alterations	Insights into the genetic regulation of development	Tresenrider et al. (2023)
Pathophysiological mechanism	Identifies genetic and transcriptional changes within retinal cells, improving understanding of DR progression	DR Research	Detailed cellular and molecular profiles of retinal cells under diabetic conditions revealed	Better strategies for preventing and treating DR	Van Hove et al. (2020)
Precision Medicine	Enhances the development of precision medicine approaches by correlating genetic mutations with functional outcomes in cells	Precision Medicine	Correlated specific genetic mutations with changes in gene expression affecting DR phenotypes	Tailored therapeutic strategies based on individual genetic profiles	Zhang X et al. (2024)

Bulk RNA-seq and scRNA-seq are often employed synergistically in multi-omics studies. While bulk RNA-seq provides a global gene expression overview, it lacks the resolution to discern gene expression at the single-cell level. Conversely, scRNA-seq excels at identifying distinct cell subpopulations but does not directly correlate these with specific phenotypes (Tan Z et al., 2023; Yu et al., 2022). By integrating both methods, researchers can comprehensively map the transcriptomic landscape, revealing cell-specific gene expression patterns and enabling the identification of cell subpopulations linked to particular phenotypes. This comprehensive approach is instrumental in elucidating disease progression and informing the development of targeted therapies (Wang, Kumar and Liu, 2021).

Integrating scRNA-seq with transcriptomics and other omics approaches provides a comprehensive framework for elucidating the cellular and molecular underpinnings of DR (Xia et al., 2023). Through multi-omic analyses, researchers have successfully identified specific cell types, signaling pathways, and potential therapeutic targets implicated in DR pathogenesis.

Mao et al. (2023) identified alternative TSS in retinal cells, underscoring the significance of 5′-UTRs in post-transcriptional regulation. Corano Scheri et al. (2023) characterized cellular composition within preretinal fibrovascular membranes, implicating endothelial, inflammatory, and stromal cells in angiogenesis and fibrosis. Becker et al. (2021) identified DEGs and pathways associated with late-stage DR, suggesting potential therapeutic targets. Investigations in diabetic models revealed dynamic changes in retinal cell populations and macroglia function, linking these cell types to retinal neurodegeneration (Li et al., 2021; Van Hove et al., 2020). Additionally, transcriptomic analysis of circulating immune cells identified Jun Proto-Oncogene D (JUND) as a key player in DR pathogenesis. Xiao et al. (2021) investigated ligand-receptor interactions in non-human primate retinas, establishing the retinal interactome and identifying TNF-α signaling in microglia activation. Deng et al. (2024) identified Müller cells as pivotal interaction hubs in the retinal microenvironment, with diverse transcriptional responses to DR.

These findings underscore the potential for developing therapeutic interventions by targeting specific cellular and molecular pathways implicated in DR. The identification of intricate cellular interactions and the discovery of novel therapeutic targets offer promising avenues for future research and clinical translation.

### 4.3 ScRNA-seq combined with metabolomic

In DR, chronic hyperglycemia disrupts the retinal metabolic microenvironment, an essential regulator of retinal cell function (Zhang J et al., 2022). Recent advancements in methodology have enabled numerous studies combining scRNA-seq with metabolomics to investigate the pathophysiology of DR using patient samples (Das et al., 2015).

Metabolomics, the comprehensive analysis of metabolites within biological samples, emerges as a promising omics tool for unraveling metabolic alterations and underlying pathophysiology in diseases such as DR. Recent studies (Eid et al., 2019; Pfeifer et al., 2023) have identified a panel of metabolites, including L-glutamine, L-lactic acid, pyruvic acid, acetic acid, L-glutamic acid, D-glucose, L-alanine, L-threonine, citrulline, L-lysine, and succinic acid, as potential biomarkers for DR, shedding light on novel disease pathways.

Serum metabolite and metabolic pathway analyses were conducted across different stages of DR in patients with type 2 diabetes mellitus. Compared to controls, DR patients from the Asian population exhibited dysregulated pathways, including arginine biosynthesis, linoleic acid metabolism, glutamate metabolism, and D-glutamine and D-glutamate metabolism. Notably, elevated levels of glutamate (Ren et al., 2024), aspartate (Zhu et al., 2018), glutamine (Yeh et al., 2020), N-acetyl-L-glutamate (Plumbly et al., 2019), and N-acetyl-L-aspartate (Ahmad et al., 2019), along with decreased levels of dihomo-gamma-linolenate, docosahexaenoic acid, and eicosatetraenoic acid, were identified as potential metabolic signatures capable of differentiating proliferative from non-proliferative DR within this population.

Advances in metabolomics technologies are illuminating critical pathways, biomarkers, and therapeutic targets for DR management (Xu et al., 2022). These insights deepen our comprehension of the disease and provide a foundation for innovative treatment strategies.

### 4.4 ScRNA-seq combined with lipidomics

Accumulating evidence underscores lipid metabolism disruption as an early hallmark in the pathogenesis of diabetic complications (Eid et al., 2019). Diverse lipid species, including glycerophospholipids (Jin et al., 2024), sphingolipids (Motloch et al., 2024), and glycerolipids (Singer et al., 2022), have been implicated as critical risk factors for DR and its associated sequelae. Conversely, reduced very long-chain ceramide levels have been linked to the onset of macroalbuminuria in diabetes. Accelerated sphingolipid catabolism, resulting in elevated glucosylceramide or glycosphingolipid concentrations, may contribute to the neuronal pathologies characteristic of DR (Ancel et al., 2023). Additionally, sphingomyelin, a lipid connected to insulin resistance and an independent predictor of cardiovascular disease, is formed by the transfer of a phosphocholine group from phosphatidylcholine to the ceramide backbone (Fernandes Silva, et al., 2023). Collectively, these findings highlight the substantial contribution of dysregulated lipid metabolism to the development of DM and its complications, although the precise lipid species driving DR remain to be fully elucidated (Pratama et al., 2022).

Lipidomic analysis, when integrated with scRNA-seq, offers a robust framework for the identification of novel lipid mediators implicated in lipid metabolism and associated biochemical processes, thereby unveiling promising avenues for disease prediction and detection (Bertelli et al., 2022). Using a quantitative metabolomics approach, Maria et al. (Bertelli et al., 2022) conducted a comparative analysis of metabolite concentrations in aqueous humor and serum from elderly individuals with and without diabetes who underwent cataract surgery.

To comprehensively characterize local metabolic alterations in activated microglia and illuminate the metabolic milieu contributing to immune responses, Lv et al. (2022) conducted integrated lipidomics and RNA profiling analyses on microglial cell line models representative of the DR microenvironment. Their findings unveiled a substantial accumulation of TAGs within activated microglia (Yousri et al., 2022). Notably, lipopolysaccharide-stimulated macrophages exhibited a similar increase in TAG synthesis (Ren et al., 2023). These observations suggest that TAG accumulation in activated microglia may serve as a potential therapeutic target for mitigating inflammation in DR.

Within the context of diabetic retinopathy, the integration of scRNA-seq with targeted metabolomic analysis has demonstrated efficacy in the precise identification of disease-associated biomarkers.

### 4.5 ScRNA-seq combined with proteomics

While scRNA-seq has yielded promising insights, its findings are limited to the transcriptomic level. A comprehensive protein expression profile is crucial for understanding cell subpopulations in DR (Fu et al., 2023). There is a strong need for single-cell proteomics, capable of detecting and quantifying over 1,000 proteins within a single mammalian cell.

High-throughput proteomics has become a valuable tool in ophthalmic research, particularly in studying DR (Starr et al., 2023). Researchers have analyzed various samples, including tears, serum, AH, VH, and retina, using techniques like mass spectrometry and liquid chromatography (Varughese and Jacob, 2023). These studies have identified key DR biomarkers, such as VEGF, IL-6, and ICAM-1. While serum and VH are directly connected to the retina, their collection can be invasive, unlike serum and tear samples (Gurel and Sheibani, 2018). Proteomic changes in these biofluids not only enhance understanding of DR pathogenesis but also hold potential for personalized medicine (Xu et al., 2014). For example, elevated plasma kallikrein in VH may suggest that standard anti-VEGF treatments are ineffective for certain patients. Wang et al. (2024) provide significant insights into the cellular and molecular mechanisms underlying PDR by identifying potential biomarkers and therapeutic targets through an integrated approach involving scRNA-seq, machine learning, AlphaFold protein structure predictions, and molecular docking techniques. Abbas et al. (2024) found scRNA-seq and proteomics are advancing our understanding of DR by enabling detailed analysis of microvesicle cargo and their roles in modulating inflammation, oxidative stress, and immune responses, thereby supporting the development of MV-based biomarkers and therapies for DR.

### 4.6 ScRNA-seq combined with other multi-omics

Ocular neovascularization can affect nearly all tissues of the eye, including the cornea, iris, retina, and choroid (Voight et al., 2010). Pathological neovascularization is a primary cause of vision loss in prevalent ocular diseases such as DR, retinopathy of prematurity, and age-related macular degeneration (Lin et al., 2023). Glycosylation, the most common covalent post-translational modification of proteins in mammalian cells, plays a significant role in angiogenesis (Zelniker et al., 2019). Emerging evidence indicates that glycosylation affects the activation, proliferation, and migration of ECs, as well as the interactions between angiogenic ECs and other cell types essential for blood vessel formation (Lin et al., 2023). Recent studies suggest that members of the galectin family, which are β-galactosidase-binding proteins, modulate angiogenesis through novel carbohydrate-based recognition systems (Ko and Moon, 2023). These systems involve interactions between the glycans on angiogenic cell surface receptors and galectins.

Recent research (Netea et al., 2020; Wimmers et al., 2021) underscores the epigenomes central role in regulating fundamental biological processes by maintaining specific chromatin states over extended periods, enabling the durable storage of gene-expression information (Khan et al., 2020). Integrating scRNA-seq with epigenomic SNP-to-gene maps, as proposed by Chen et al. (2023b), provides a comprehensive framework to identify cell types and processes affected by genetic variants, offering new insights into DR pathogenesis and potential therapeutic targets. GWAS have identified genetic variants associated with DR, helping to clarify disease susceptibility (Skol et al., 2020). Researchers have assessed DR risk genes across various cell types to identify those most vulnerable to DR, emphasizing the sensitivity of bipolar, amacrine, and Müller glial cells. Since many DR-related variants lie in noncoding regions, their interpretation using scRNA-seq alone is limited (Jagadeesh et al., 2022). By linking GWAS data with scRNA-seq and tissue-specific enhancer-gene mappings, researchers can detect tissue-specific SNP-to-gene associations that reveal differences in disease heritability across cell types. Furthermore, analyzing disease-specific cell states in both healthy and diseased tissues highlights cell-type changes linked to DR. Finally, non-negative matrix factorization enables the identification of cellular process programs—such as MAPK signaling—that extend across cell types, deepening our understanding of DR pathogenesis at cellular and molecular levels.

Spatial gene expression is an advancing technology that has recently seen the development of commercial systems capable of achieving true single-cell or subcellular resolution of gene expression within a spatial transcriptomics (Voigt et al., 2023). However, these systems often rely on probe-based hybridization, which requires the design of custom gene panels (Feng et al., 2024). Unbiased sequencing-based platforms are also progressing, with improvements in spot resolution approaching near single-cell levels (Marneros, 2023). This study demonstrates the utility of spatial technology in investigating gene expression within focal pathological areas and along regional gradients in the heterogeneous retina, RPE, and choroid. Ongoing research, coupled with technological advancements, will continue to enhance our understanding of the pathogenic mechanisms underlying macular neovascularization and other regional retinal diseases (Chen X et al., 2024). The insights gained from this powerful technology may inform the development of future targeted therapies, potentially reducing vision loss associated with DR.

High-throughput technologies and omics data, such as glycolipidosis, proteomics, and spatial transcriptomics analysis, have advanced our understanding of DR by identifying risk factors and developing novel biomarkers (Tolentino et al., 2023). By integrating these data through bioinformatics, researchers gain comprehensive insights into the susceptibility genes, mechanistic pathways, and disease stage markers of DR (Kakihara et al., 2023). However, scRNA analyses of glycolipidosis, epigenomes and spatial transcriptomics in DR remain underreported.

## 5 The advantages and challenges of scRNA-seq with multi-omics in DR research

### 5.1 The advantages of scRNA-seq

#### 5.1.1 Computational resources: Databases of interacting proteins

CellPhone DB, a newly established public resource, comprehensively catalogs ligands, receptors, and their interactions to facilitate the analysis of cell-cell communication molecules (Chen et al., 2022). This framework leverages single-cell transcriptomic data to quantify ligand and receptor expression across diverse cell types, identifying cell-type-specific ligand-receptor pairs through empirical shuffling (Xu et al., 2023a). Unlike many databases, CellPhone DB accurately represents heteromeric complexes by considering the subunit architecture of both ligands and receptors (Qin et al., 2023).

The latest CellPhone DB update incorporates enhanced functionalities, facilitating the inclusion of novel interacting molecules and streamlining analyses of extensive datasets (Cabrera et al., 2022). These advancements significantly improve the annotation of intricate ligand-receptor interactions using scRNA-seq data. Future research integrating scRNA-seq with CyTOF and CellPhone DB holds the potential to uncover novel disease mechanisms (Yang et al., 2023). Additionally, optimization methods like SoptSC and tools from the FANTOM5 project offer alternative approaches to deciphering cell-cell relationships from single-cell data (Sørensen et al., 2023). This approach enables a more comprehensive and precise investigation of previously unknown cell-cell interactions, offering opportunities for further validation beyond traditional methods. In this context, we outline the cell-cell communication observed in DR using scRNA-seq from prior studies, highlighting the enhanced interactions between retinal cells under hyperglycemic conditions and the intercellular crosstalk identified among cells within fibrovascular membranes (FVMs) (Figure 4).

#### 5.1.2 Modeling and assessing predicted interaction networks

Single-cell multi-omics methodologies provide a holistic perspective on cellular expression, function, and identity, though a complete comprehension of these intricate systems remains elusive (Pelka et al., 2021). While a substantial portion of research has focused on utilizing scRNA-seq to elucidate communication networks through ligand-receptor interactions within the complex DR microenvironment (Luo et al., 2023), recent endeavors by Chen and Mar have delved into evaluating the efficacy of single-cell network modeling techniques in uncovering established interaction networks within single-cell datasets (Zhang et al., 2021). These methods, including SCENIC, SCODE, and PIDC, employ disparate approaches, respectively, leveraging co-expression networks with bioinformatics knowledge, ordinary differential equations, and mutual information-based strategies (Jumeau et al., 2018).

A comparative analysis of existing methods revealed significant limitations in accurately predicting network structures from single-cell expression data (Saint-Antoine and Singh, 2020). While these approaches offer valuable insights, they consistently failed to achieve high predictive accuracy, and the resulting networks exhibited substantial variability across different methodologies (Deligiannidis et al., 2023; Karyotaki et al., 2021). This inconsistency poses a considerable challenge for accurately predicting cell-cell communication networks, as the choice of modeling approach can substantially influence the outcomes (Hussain et al., 2021).

Although this review does not delve deeply into the intricacies of these modeling methodologies, a comprehensive exploration of their predictive capabilities is indispensable for a thorough understanding of their potential applications and limitations.

#### 5.1.3 Validating causal relationships

Integrating single-cell multi-omics data with perturbation experiments, such as RNA interference or CRISPR-Cas9, offers a robust approach for validating causal regulatory programs (Bowling et al., 2020). Recent advancements in high-throughput gene technologies, exemplified by Perturb-seq, have merged CRISPR/Cas9-mediated gene perturbation with single-cell sequencing (Li et al., 2023), demonstrating comparable efficacy in identifying causal relationships to traditional RNAi or CRISPR-Cas9-based gene activation/deletion methods while exhibiting reduced invasiveness (Raj et al., 2018).

Advances in single-cell technologies are poised to expand the scope of cellular parameters measurable at the individual cell level (Choi, 2020). Concurrent multi-modal profiling within single cells holds great promise for predicting drug sensitivities in retinal cells, potentially obviating the need for extensive *in vivo* or *in vitro* experimentation (Pei et al., 2020). The application of these techniques within the framework of single-cell multi-omics studies on DR has the potential to significantly enhance our comprehension of the disease and accelerate the development of targeted therapeutic interventions (Bucheli et al., 2021; Wang et al., 2020).

### 5.2 The challenges in scRNA-seq

#### 5.2.1 Cell dissociation protocols

The mammalian retina is a complex tissue composed of interconnected neurons, glial cells, and photoreceptors (Ye et al., 2020). Achieving high cell viability and quality in scRNA-seq studies of DR is particularly challenging due to the retina’s delicate nature (Lin et al., 2024). The requisite process of cell dissociation to isolate individual cells for sequencing can induce cellular stress and damage, potentially distorting gene expression profiles and introducing spurious artifacts that obscure authentic biological signals (Yao et al., 2022).

Preserving cell viability and minimizing stress during dissociation are paramount for accurately capturing gene expression profiles that reflect *in vivo* conditions (Raj et al., 2018). Such precision is indispensable for elucidating the cellular and molecular underpinnings of DR. Photoreceptors, characterized by their delicate structure, are susceptible to both enzymatic and mechanical dissociation, resulting in RNA leakage from compromised cells (VanHorn and Morris, 2021). Notably, disease states such as DR may render cells even more fragile during isolation.

Obtaining high-quality, viable retinal cells is paramount for accurately identifying cell types most affected by DR. Data derived from damaged or stressed cells can lead to unreliable results, potentially misrepresenting cell vulnerability or resilience (Peng et al., 2020). Precise gene expression profiling is essential for discovering biomarkers and therapeutic targets. Artifacts introduced by cell stress or damage can generate false positives or negatives, impeding the identification of meaningful biomarkers and targets (Wang et al., 2023). Moreover, maintaining high cell viability and quality is crucial for the reproducibility of scRNA-seq studies. Consistent and reliable data across multiple studies and laboratories are fundamental for validating findings and advancing our understanding of DR (Beltrami et al., 2022).

#### 5.2.2 Spatial and temporal information

Human NR are light-sensitive tissues distinguished by a complex spatial and cellular architecture (Lukowski et al., 2019). While single-cell omics technologies offer a comprehensive characterization of cellular types and states, the intricate functionality of tissues is profoundly shaped by the spatial disposition of these cells (Quadrato et al., 2017). Despite its capacity to analyze cellular interactions, scRNA-seq is unable to delineate cell localization or spatial context (Lancaster et al., 2013). Cells residing within local microenvironments exert specific physiological roles through juxtacrine and paracrine signaling, as well as intercellular interactions (Cohen et al., 2021).

The retina’s precise spatial organization is fundamental to its function, and disruptions caused by infections, inflammation, or injuries can significantly alter this architecture, impacting visual acuity (Deng et al., 2024). Understanding these organizational changes is crucial for diagnosing and treating retinal diseases. Traditional histopathological techniques, complemented by *in situ* hybridization and immunohistochemistry (Md Pauzi et al., 2021), examine tissue architecture and molecular markers. However, these methods are limited in their ability to comprehensively characterize the retina’s molecular landscape, as they can only assess a restricted number of transcripts or proteins per experiment.

Spatial context is paramount in comprehending complex tissue function, as cellular positioning and interactions within microenvironments significantly influence tissue behavior (Fang et al., 2022). Spatial transcriptomics offers unprecedented insights into disease progression by mapping cellular and tissue-level changes across time and space (Cao et al., 2023). This technology enables the identification of disease-specific microenvironments within the retina, revealing how local cellular contexts contribute to disease pathology (Liu et al., 2023). By elucidating the spatial distribution of gene expression alterations, researchers can uncover novel therapeutic targets and develop treatments precisely targeted to affected tissue regions (Geng et al., 2021).

#### 5.2.3 Intercellular communication

**FIGURE 5:**
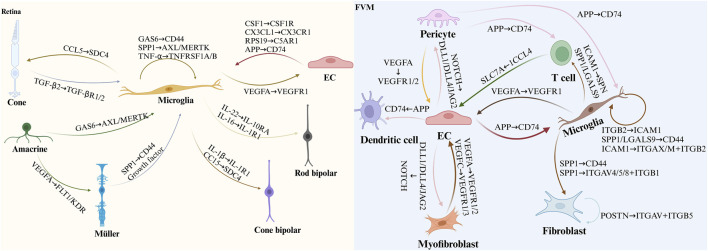
Illustrates cell-cell communication in DR as uncovered by scRNA-seq. The figure depicts ligand-receptor interactions associated with DR, based on findings from previous scRNA-seq studies. In the diagram, the arrowhead points to the receptor protein and the corresponding cell expressing it, while the arrowtail indicates the ligand protein and the cell producing it. This representation sheds light on the disrupted communication networks in diabetic retinas and the intercellular signaling within FVM, which may contribute to the pathogenesis of DR.

The retina’s unique architecture, characterized by complex cellular interactions and signaling pathways, distinguishes it from other tissues, including the brain (Huang et al., 2023). This intricate organization has historically limited investigations into cell-cell communication within the retina, particularly in the context of DR development (Li et al., 2023; Lou et al., 2022; Yi et al., 2021). Nevertheless, comprehending these intercellular relationships is indispensable for elucidating the mechanisms underlying retinal degeneration (Figure 5).

Normally, the retina is protected from systemic circulation by the blood-retinal barrier, composed of the RPE in the outer retina and ECs, pericytes, and astrocyte end-feet in the inner retina (Voigt et al., 2019). While various retinal cell types exhibit differential susceptibility to DR in mice, Müller glia and clusters of rods, rod BCs, cones, and vascular ECs are prominently involved in proliferative DR pathogenesis (Mullin et al., 2023; Voigt et al., 2021b). The precise role and activation mechanisms of immune cells in DR remain to be fully elucidated (Nishikawa and Koyama, 2021).

Exploring key retinal cell types and their interactions holds promise for developing novel therapeutic approaches not only for DR but also for other neurodegenerative diseases affecting the central nervous system (Niu et al., 2021). In both diabetic and healthy mice, RGCs, vascular ECs, and Müller glia exhibit significant activity (Tresenrider et al., 2023; Yao et al., 2023). Notably, microglial involvement in retinal cell-cell communication networks is markedly increased in DR mice compared to controls (Parikh et al., 2023). These findings emphasize the critical role of understanding cellular interactions and the functions of various cell types in DR pathogenesis (Beilke et al., 1991). Such knowledge could lead to the identification of promising therapeutic targets for disease management and visual function preservation (Tan et al., 2021).

## 6 Future outlook

Recent advancements in scRNA-seq have significantly deepened our understanding of DR by providing comprehensive molecular profiles of retinal cell types, including photoreceptors, ECs, and pericytes, integral to blood-retinal barrier integrity (Chen et al., 2024; Rangasamy et al., 2020; Sun et al., 2021; Zhan, 2023). Profiling these cells reveals critical pathways for their survival and function, identifying potential therapeutic targets. Data-sharing platforms like the Single Cell Portal and Spectacle database facilitate the integration of diverse datasets, aiding in elucidating DR pathogenesis (Corano Scheri et al., 2023). Addressing data heterogeneity through tools such as SIDA and Harmony is imperative for analyzing large-scale scRNA-seq data (Paik et al., 2020). While current studies predominantly offer gene expression snapshots, longitudinal studies and spatial transcriptomics are essential for comprehending DR dynamics and evaluating therapeutic efficacy (Fadakar et al., 2024). Liquid biopsy, particularly exosome analysis, shows promise for early detection. Integrating multi-omics approaches, encompassing transcriptomic, proteomic, and metabolomic data, will provide a comprehensive understanding of DR mechanisms and inform the development of effective treatments (Manochkumar et al., 2023; Xu et al., 2023).

## 7 Conclusion

ScRNA-seq is a revolutionary technology enabling transcriptomic analysis at the individual cell level, providing unprecedented insights into the complex cellular heterogeneity of DR. By revealing cellular landscapes, identifying biomarkers, and informing treatment strategies, scRNA-seq is accelerating the development of precision medicine for DR. As technology advances, integration with other omics approaches, particularly spatial gene expression, holds the potential to unlock deeper understanding of gene regulatory networks and disease mechanisms. While challenges remain, the synergy between laboratory research and clinical applications promises to optimize patient outcomes through the development of targeted interventions.

## Glossary

ACER2: Alkaline Ceramidase 2
AD: Alzheimer’s disease
AEBP1: Adipocyte Enhancer-Binding Protein 1
APP: Amyloid-beta precursor protein
BC: Bipolar cell
BCs: Bipolar cells
BRB: Blood-retina barrier
Col1a1: Collagen Type I Alpha 1 Chain
DEGs: Differentially expressed genes
DEP: Dielectrophoretic
DME: Diabetic macular edema
DN: Diabetic nephropathy
DRECs: Diabetic Retinal Endothelial Cells
EC: Endothelial cell
FACS: Fluorescence-Activated Cell Sorting
FVM: Fibrovascular membrane
**GLP**-1: Glucagon-like peptide-1
**GLP**-1 **RA**: Glucagon-like peptide-1 receptor agonist
GWAS: Population-based genome-wide association studies
ICAM1: Intercellular Adhesion Molecule 1
IEG: Immediate early gene
INL: Inner nuclear layer
IPL: Inner plexiform layer
JUND: JunD Proto-Oncogene, AP-1 Transcription Factor Subunit
LCM: Laser Capture Microdissection
MACS: Magnetic-Activated Cell Sorting
MC: Mesangial cell
NDR: Non-diabetic retinopathy
NK: Natural killer
NPDR-NDME: Non-proliferative diabetic retinopathy without diabetic macular edema
NR: Neural retina
OIR: Oxygen-induced retinopathy
ONL: Outer nuclear layer
OPL: Outer plexiform layer
PBMC: Peripheral blood mononuclear cell
PDE6G: Phosphodiesterase 6G
PDR: Proliferative diabetic retinopathy
POSTN: Periostin
RBC: Retinal bipolar cell
RGC: Retinal Ganglion Cell
RLBP1: Retinaldehyde Binding Protein 1
RNAi: RNA interference
RPCs: Retinal pericytes
RPE: Retinal pigment epithelium
SCENIC: Single-cell regulatory network inference and clustering
ST: Spatial transcriptomics
STZ: Streptozotocin
DM: Diabetes mellitus
TAG: Triglycerides
TSS: Transcription start sites
VEGFR1: Vascular endothelial growth factor receptor

## References

[B1] Abbas A. Almaghrbi H. Giordo R. Zayed H. Pintus G. (2024). Pathogenic mechanisms, diagnostic, and therapeutic potential of microvesicles in diabetes and its complications. Archives Biochem. Biophysics 761, 110168. 10.1016/j.abb.2024.110168 39349130

[B2] Ahmad A. F. Dwivedi G. O'Gara F. Caparros-Martin J. Ward N. C. (2019). The gut microbiome and cardiovascular disease: current knowledge and clinical potential. Am. J. Physiology. Heart Circulatory Physiology 317 (5), H923–H938. 10.1152/ajpheart.00376.2019 31469291

[B3] Ancel P. Martin J. C. Doukbi E. Houssays M. Gascon P. Righini M. (2023). Untargeted multiomics approach coupling lipidomics and metabolomics profiling reveals new insights in diabetic retinopathy. Int. J. Mol. Sci. 24 (15), 12053. 10.3390/ijms241512053 37569425 PMC10418671

[B4] Ashraf M. Sampani K. Rageh A. Silva P. S. Aiello L. P. Sun J. K. (2020). Interaction between the distribution of diabetic retinopathy lesions and the association of optical coherence tomography angiography scans with diabetic retinopathy severity. JAMA Ophthalmol. 138 (12), 1291–1297. 10.1001/jamaophthalmol.2020.4516 33119083 PMC7596681

[B5] Bawa G. Liu Z. Yu X. Qin A. Sun X. (2022). Single-cell RNA sequencing for plant research: insights and possible benefits. Int. J. Mol. Sci. 23 (9), 4497. 10.3390/ijms23094497 35562888 PMC9100049

[B6] Becker K. Klein H. Simon E. Viollet C. Haslinger C. Leparc G. (2021). In-depth transcriptomic analysis of human retina reveals molecular mechanisms underlying diabetic retinopathy. Sci. Rep. 11 (1), 10494. 10.1038/s41598-021-88698-3 34006945 PMC8131353

[B7] Beilke M. A. In D. R. Gravell M. Hamilton R. S. Mora C. A. Leon-Monzon M. (1991). *In situ* hybridization detection of HTLV-I RNA in peripheral blood mononuclear cells of TSP/HAM patients and their spouses. J. Med. Virology 33 (1), 64–71. 10.1002/jmv.1890330113 1849984

[B8] Beltrami E. J. Brown A. C. Salmon P. J. M. Leffell D. J. Ko J. M. Grant-Kels J. M. (2022). Artificial intelligence in the detection of skin cancer. J. Am. Acad. Dermatology 87 (6), 1336–1342. 10.1016/j.jaad.2022.08.028 35998842

[B9] Beneyto-Calabuig S. Merbach A. K. Kniffka J.-A. Antes M. Szu-Tu C. Rohde C. (2023). Clonally resolved single-cell multi-omics identifies routes of cellular differentiation in acute myeloid leukemia. Cell Stem Cell 30 (5), 706–721.e8. 10.1016/j.stem.2023.04.001 37098346

[B10] Bertelli P. M. Pedrini E. Hughes D. McDonnell S. Pathak V. Peixoto E. (2022). Long term high glucose exposure induces premature senescence in retinal endothelial cells. Front. Physiology 13, 929118. 10.3389/fphys.2022.929118 PMC9459081 36091370

[B11] Blanchard J. W. Akay L. A. Davila-Velderrain J. von Maydell D. Mathys H. Davidson S. M. (2022). APOE4 impairs myelination via cholesterol dysregulation in oligodendrocytes. Nature 611 (7937), 769–779. 10.1038/s41586-022-05439-w 36385529 PMC9870060

[B12] Bowling S. Sritharan D. Osorio F. G. Nguyen M. Cheung P. Rodriguez-Fraticelli A. (2020). An engineered CRISPR-cas9 mouse line for simultaneous readout of lineage histories and gene expression profiles in single cells. Cell 181 (6), 1410–1422. 10.1016/j.cell.2020.04.048 32413320 PMC7529102

[B13] Bryl A. Mrugacz M. Falkowski M. Zorena K. (2022). The effect of diet and lifestyle on the course of diabetic retinopathy-A review of the literature. Nutrients 14 (6), 1252. 10.3390/nu14061252 35334909 PMC8955064

[B14] Bucheli O. T. M. Sigvaldadóttir I. Eyer K. (2021). Measuring single-cell protein secretion in immunology: technologies, advances, and applications. Eur. J. Immunol. 51 (6), 1334–1347. 10.1002/eji.202048976 33734428 PMC8252417

[B15] Cabrera J. P. Camino-Willhuber G. Guiroy A. Carazzo C. A. Gagliardi M. Joaquim A. F. (2022). Vertebral augmentation plus short-segment fixation versus vertebral augmentation alone in Kümmell's disease: a systematic review and meta-analysis. Neurosurg. Rev. 45 (2), 1009–1018. 10.1007/s10143-021-01661-8 34596773

[B16] Cao N. Ouyang H. Zhang X. Xu Y. Li J. Chen Y. (2023). Integration of scRNA-Seq and bulk RNA-Seq uncover perturbed immune cell types and pathways of Kawasaki disease. Front. Immunol. 14, 1259353. 10.3389/fimmu.2023.1259353 37841239 PMC10568768

[B17] Chen B. Zou J. Xie L. Cai Y. Li B. Tan W. (2024a). WNT-inhibitory factor 1-mediated glycolysis protects photoreceptor cells in diabetic retinopathy. J. Transl. Med. 22 (1), 245. 10.1186/s12967-024-05046-5 38448948 PMC10918886

[B18] Chen K. Wang Y. Huang Y. Liu X. Tian X. Yang Y. (2023b). Cross-species scRNA-seq reveals the cellular landscape of retina and early alterations in type 2 diabetes mice. Genomics 115 (4), 110644. 10.1016/j.ygeno.2023.110644 37279838

[B19] Chen S. Guo D. Zhu Y. Xiao S. Xie J. Zhang Z. (2024b). Amyloid β oligomer induces cerebral vasculopathy via pericyte-mediated endothelial dysfunction. Alzheimer's Res. & Ther. 16 (1), 56. 10.1186/s13195-024-01423-w 38475929 PMC10935813

[B20] Chen Y. Wang H. Yang Q. Zhao W. Chen Y. Ni Q. (2022). Single-cell RNA landscape of the osteoimmunology microenvironment in periodontitis. Theranostics 12 (3), 1074–1096. 10.7150/thno.65694 35154475 PMC8771561

[B21] Cheng Y Y. Zhang M. Xu R. Fu L. Xue M. Xu C. (2024). p53 accelerates endothelial cell senescence in diabetic retinopathy by enhancing FoxO3a ubiquitylation and degradation via UBE2L6. Exp. Gerontol. 188, 112391. 10.1016/j.exger.2024.112391 38437929

[B22] Chen X X. Qin X. Bai W. Ren J. Yu Y. Nie H. (2024). Kavain alleviates choroidal neovascularization via decreasing the activity of the HIF-1α/VEGF-A/VEGFR2 signaling pathway and inhibiting inflammation. Adv. Pharm. Bull. 14 (2), 469–482. 10.34172/apb.2024.036 39206403 PMC11347728

[B23] Choi J. R. (2020). Advances in single cell technologies in immunology. BioTechniques 69 (3), 226–236. 10.2144/btn-2020-0047 32777935

[B24] Cochain C. Vafadarnejad E. Arampatzi P. Pelisek J. Winkels H. Ley K. (2018). Single-cell RNA-seq reveals the transcriptional landscape and heterogeneity of aortic macrophages in murine atherosclerosis. Circulation Res. 122 (12), 1661–1674. 10.1161/CIRCRESAHA.117.312509 29545365

[B25] Cohen Y. C. Zada M. Wang S.-Y. Bornstein C. David E. Moshe A. (2021). Identification of resistance pathways and therapeutic targets in relapsed multiple myeloma patients through single-cell sequencing. Nat. Med. 27 (3), 491–503. 10.1038/s41591-021-01232-w 33619369 PMC7612793

[B26] Corano Scheri K. Lavine J. A. Tedeschi T. Thomson B. R. Fawzi A. A. (2023). Single-cell transcriptomics analysis of proliferative diabetic retinopathy fibrovascular membranes reveals AEBP1 as fibrogenesis modulator. JCI Insight 8 (23), e172062. 10.1172/jci.insight.172062 37917183 PMC10896003

[B27] Das A. McGuire P. G. Rangasamy S. (2015). Diabetic macular edema: pathophysiology and novel therapeutic targets. Ophthalmology 122 (7), 1375–1394. 10.1016/j.ophtha.2015.03.024 25935789

[B28] Deligiannidis K. M. Meltzer-Brody S. Maximos B. Peeper E. Q. Freeman M. Lasser R. (2023). Zuranolone for the treatment of postpartum depression. Am. J. Psychiatry 180 (9), 668–675. 10.1176/appi.ajp.20220785 37491938

[B29] Deng X. Mo Y. Zhu X. (2024). Deciphering Müller cell heterogeneity signatures in diabetic retinopathy across species: an integrative single-cell analysis. Eur. J. Med. Res. 29 (1), 265. 10.1186/s40001-024-01847-y 38698486 PMC11067085

[B30] De Rop F. V. Ismail J. N. Bravo González-Blas C. Hulselmans G. J. Flerin C. C. Janssens J. (2022). Hydrop enables droplet-based single-cell ATAC-seq and single-cell RNA-seq using dissolvable hydrogel beads. ELife 11, e73971. 10.7554/eLife.73971 35195064 PMC8993220

[B211] Ding S. Chen X. Shen K. (2020). Single-cell RNA sequencing in breast cancer: understanding tumor heterogeneity and paving roads to individualized therapy. Cancer Commun (Lond) 40 (8), 329–344. 10.1002/cac2.12078 32654419 PMC7427308

[B31] Eid S. Sas K. M. Abcouwer S. F. Feldman E. L. Gardner T. W. Pennathur S. (2019). New insights into the mechanisms of diabetic complications: role of lipids and lipid metabolism. Diabetologia 62 (9), 1539–1549. 10.1007/s00125-019-4959-1 31346658 PMC6679814

[B32] Fadakar K. Rahmani S. Tedeschi T. Lavine J. A. Fawzi A. A. (2024). Short term effect of pre-operative anti-VEGF on angiogenic and fibrotic profile of fibrovascular membranes of proliferative diabetic retinopathy. Investigative Ophthalmol. & Vis. Sci. 65 (4), 37. 10.1167/iovs.65.4.37 PMC11044842 38652648

[B33] Fan Y. Pedersen O. (2021). Gut microbiota in human metabolic health and disease. Nat. Rev. Microbiol. 19 (1), 55–71. 10.1038/s41579-020-0433-9 32887946

[B34] Fang Z. Li J. Cao F. Li F. (2022). Integration of scRNA-seq and bulk RNA-seq reveals molecular characterization of the immune microenvironment in acute pancreatitis. Biomolecules 13 (1), 78. 10.3390/biom13010078 36671463 PMC9855877

[B35] Feng D.-C. Zhu W.-Z. Wang J. Li D.-X. Shi X. Xiong Q. (2024). The implications of single-cell RNA-seq analysis in prostate cancer: unraveling tumor heterogeneity, therapeutic implications and pathways towards personalized therapy. Mil. Med. Res. 11 (1), 21. 10.1186/s40779-024-00526-7 38605399 PMC11007901

[B36] Fernandes Silva L. Hokkanen J. Vangipurapu J. Oravilahti A. Laakso M. (2023). Metabolites as risk factors for diabetic retinopathy in patients with type 2 diabetes: a 12-year follow-up study. J. Clin. Endocrinol. Metabolism 109 (1), 100–106. 10.1210/clinem/dgad452 PMC10735554 37560996

[B37] Fu Q. Jiang H. Qian Y. Lv H. Dai H. Zhou Y. (2023). Single-cell RNA sequencing combined with single-cell proteomics identifies the metabolic adaptation of islet cell subpopulations to high-fat diet in mice. Diabetologia 66 (4), 724–740. 10.1007/s00125-022-05849-5 36538064 PMC9765371

[B38] Gan G. Lin S. Luo Y. Zeng Y. Lu B. Zhang R. (2024). Unveiling the oral-gut connection: chronic apical periodontitis accelerates atherosclerosis via gut microbiota dysbiosis and altered metabolites in apoE-/- Mice on a high-fat diet. Int. J. Oral Sci. 16 (1), 39. 10.1038/s41368-024-00301-3 38740741 PMC11091127

[B39] Gao N. Hao S. Huang G. Hao W. Su L. (2022). The integrated transcriptome bioinformatics analysis identifies key genes and cellular components for proliferative diabetic retinopathy. PLoS One 17 (11), e0277952. 10.1371/journal.pone.0277952 36409751 PMC9678275

[B40] Geng Z. Tao Y. Zheng F. Wu L. Wang Y. Wang Y. (2021). Altered monocyte subsets in kawasaki disease revealed by single-cell RNA-sequencing. J. Inflamm. Res. 14, 885–896. 10.2147/JIR.S293993 33758528 PMC7981157

[B41] Gérard A. Woolfe A. Mottet G. Reichen M. Castrillon C. Menrath V. (2020). Author Correction: high-throughput single-cell activity-based screening and sequencing of antibodies using droplet microfluidics. Nat. Biotechnol. 38 (6), 756. 10.1038/s41587-020-0563-7 32444853

[B42] Ghita L. Yao Z. Xie Y. Duran V. Cagirici H. B. Samir J. (2023). Global and cell type-specific immunological hallmarks of severe dengue progression identified via a systems immunology approach. Nat. Immunol. 24 (12), 2150–2163. 10.1038/s41590-023-01654-3 37872316 PMC10863980

[B43] Golubnitschaja O. Polivka J. Potuznik P. Pesta M. Stetkarova I. Mazurakova A. (2024). The paradigm change from reactive medical services to 3PM in ischemic stroke: a holistic approach utilising tear fluid multi-omics, mitochondria as a vital biosensor and AI-based multi-professional data interpretation. EPMA J. 15 (1), 1–23. 10.1007/s13167-024-00356-6 38463624 PMC10923756

[B44] Gui F. You Z. Fu S. Wu H. Zhang Y. (2020). Endothelial dysfunction in diabetic retinopathy. Front. Endocrinol. (Lausanne) 11, 591. 10.3389/fendo.2020.00591 33013692 PMC7499433

[B45] Guo X. Ning J. Chen Y. Liu G. Zhao L. Fan Y. (2024). Recent advances in differential expression analysis for single-cell RNA-seq and spatially resolved transcriptomic studies. Briefings Funct. Genomics 23 (2), 95–109. 10.1093/bfgp/elad011 37022699

[B46] Gurel Z. Sheibani N. (2018). O-Linked β-N-acetylglucosamine (O-GlcNAc) modification: a new pathway to decode pathogenesis of diabetic retinopathy. Clin. Sci. Lond. Engl. 1979 132 (2), 185–198. 10.1042/CS20171454 PMC6016849 29352075

[B47] He H. Suryawanshi H. Morozov P. Gay-Mimbrera J. Del Duca E. Kim H. J. (2020). Single-cell transcriptome analysis of human skin identifies novel fibroblast subpopulation and enrichment of immune subsets in atopic dermatitis. J. Allergy Clin. Immunol. 145 (6), 1615–1628. 10.1016/j.jaci.2020.01.042 32035984

[B48] He X. Wen S. Tang X. Wen Z. Zhang R. Li S. (2024). Glucagon-like peptide-1 receptor agonists rescued diabetic vascular endothelial damage through suppression of aberrant STING signaling. Acta Pharm. Sin. B 14 (6), 2613–2630. 10.1016/j.apsb.2024.03.011 38828140 PMC11143538

[B49] Herrera-Luis E. Ortega V. E. Ampleford E. J. Sio Y. Y. Granell R. de Roos E. (2022). Multi-ancestry genome-wide association study of asthma exacerbations. Pediatr. Allergy Immunol. Official Publ. Eur. Soc. Pediatr. Allergy Immunol. 33 (6), e13802. 10.1111/pai.13802 PMC9671132 35754128

[B213] Heumos L. Schaar A. C. Lance C. Litinetskaya A. Drost F. Zappia L. (2023). Best practices for single-cell analysis across modalities. Nat. Rev. Gene. 24 (8), 550–572. 10.1038/s41576-023-00586-w PMC10066026 37002403

[B50] Hu Y. Wang X. Hu B. Mao Y. Chen Y. Yan L. (2019). Dissecting the transcriptome landscape of the human fetal neural retina and retinal pigment epithelium by single-cell RNA-seq analysis. PLoS Biol. 17 (7), e3000365. 10.1371/journal.pbio.3000365 31269016 PMC6634428

[B51] Hu Z. Mao X. Chen M. Wu X. Zhu T. Liu Y. (2022). Single-cell transcriptomics reveals novel role of microglia in fibrovascular membrane of proliferative diabetic retinopathy. Diabetes 71 (4), 762–773. 10.2337/db21-0551 35061025

[B52] Hu Z. Wang J. Pan T. Li X. Tao C. Wu Y. (2023). The exosome-transmitted lncRNA LOC100132249 induces endothelial dysfunction in diabetic retinopathy. Diabetes 72 (9), 1307–1319. 10.2337/db22-0435 37347724

[B53] Huang L. Li R. Ye L. Zhang S. Tian H. Du M. (2023). Deep Sc-RNA sequencing decoding the molecular dynamic architecture of the human retina. Sci. China. Life Sci. 66 (3), 496–515. 10.1007/s11427-021-2163-1 36115892

[B54] Hussain Z. Rahim M. A. Jan N. Shah H. Rawas-Qalaji M. Khan S. (2021). Cell membrane cloaked nanomedicines for bio-imaging and immunotherapy of cancer: improved pharmacokinetics, cell internalization and anticancer efficacy. J. Control. Release Official J. Control. Release Soc. 335, 130–157. 10.1016/j.jconrel.2021.05.018 34015400

[B55] Ising M. Maccarrone G. Brückl T. Scheuer S. Hennings J. Holsboer F. (2019). FKBP5 gene expression predicts antidepressant treatment outcome in depression. Int. J. Mol. Sci. 20 (3), 485. 10.3390/ijms20030485 30678080 PMC6387218

[B56] Jagadeesh K. A. Dey K. K. Montoro D. T. Mohan R. Gazal S. Engreitz J. M. (2022). Identifying disease-critical cell types and cellular processes by integrating single-cell RNA-sequencing and human genetics. Nat. Genet. 54 (10), 1479–1492. 10.1038/s41588-022-01187-9 36175791 PMC9910198

[B57] Jin Y. Yong S. Ke S. Zhang C. Liu Y. Wang J. (2024). Deep learning assisted fluid volume calculation for assessing anti-vascular endothelial growth factor effect in diabetic macular edema. Heliyon 10 (8), e29775. 10.1016/j.heliyon.2024.e29775 38699726 PMC11063453

[B58] Jovic D. Liang X. Zeng H. Lin L. Xu F. Luo Y. (2022). Single-cell RNA sequencing technologies and applications: a brief overview. Clin. Transl. Med. 12 (3), e694. 10.1002/ctm2.694 35352511 PMC8964935

[B59] Jumeau R. Nguyen-Tan P. F. Bahig H. Liem X. Lambert L. Schmittbuhl M. (2018). Pre-irradiation dental care: ready-to-use templates for oropharyngeal cancers. Rep. Pract. Oncol. Radiotherapy J. Gt. Cancer Cent. Poznan Pol. Soc. Radiat. Oncol. 23 (4), 270–275. 10.1016/j.rpor.2018.06.007 PMC6078107 30090026

[B60] Kakihara S. Matsuda Y. Hirabayashi K. Imai A. Iesato Y. Sakurai T. (2023). Role of adrenomedullin 2/intermedin in the pathogenesis of neovascular age-related macular degeneration. Laboratory Investigation; a J. Tech. Methods Pathology 103 (4), 100038. 10.1016/j.labinv.2022.100038 36870288

[B61] Kang Q. Yang C. (2020). Oxidative stress and diabetic retinopathy: molecular mechanisms, pathogenetic role and therapeutic implications. Redox Biol. 37, 101799. 10.1016/j.redox.2020.101799 33248932 PMC7767789

[B62] Karyotaki E. Efthimiou O. Miguel C. Bermpohl F. M. G. Furukawa T. A. Cuijpers P. (2021). Internet-based cognitive behavioral therapy for depression: a systematic review and individual patient data network meta-analysis. JAMA Psychiatry 78 (4), 361–371. 10.1001/jamapsychiatry.2020.4364 33471111 PMC8027916

[B63] Khan N. Paterson A. D. Roshandel D. Raza A. Ajmal M. Waheed N. K. (2020). Association of IGF1 and VEGFA polymorphisms with diabetic retinopathy in Pakistani population. Acta Diabetol. 57 (2), 237–245. 10.1007/s00592-019-01407-5 31473834

[B64] Kim U. S. Mahroo O. A. Mollon J. D. Yu-Wai-Man P. (2021). Retinal ganglion cells-diversity of cell types and clinical relevance. Front. Neurology 12, 661938. 10.3389/fneur.2021.661938 PMC8175861 34093409

[B65] Kinuthia U. M. Wolf A. Langmann T. (2020). Microglia and inflammatory responses in diabetic retinopathy. Front. Immunol. 11, 564077. 10.3389/fimmu.2020.564077 33240260 PMC7681237

[B66] Kleino I. Frolovaitė P. Suomi T. Elo L. L. (2022). Computational solutions for spatial transcriptomics. Comput. Struct. Biotechnol. J. 20, 4870–4884. 10.1016/j.csbj.2022.08.043 36147664 PMC9464853

[B67] Ko J. Moon S. J. (2023). Risk of diabetic retinopathy between sodium-glucose cotransporter-2 inhibitors and glucagon-like peptide-1 receptor agonists. Diabetes Metab. J. 47, 394–404. 10.4093/dmj.2023.0164 36872060 PMC10244193

[B68] Koh A. Bäckhed F. (2020). From association to causality: the role of the gut microbiota and its functional products on host metabolism. Mol. Cell 78 (4), 584–596. 10.1016/j.molcel.2020.03.005 32234490

[B69] Laboissonniere L. A. Goetz J. J. Martin G. M. Bi R. Lund T. J. S. Ellson L. (2019). Molecular signatures of retinal ganglion cells revealed through single cell profiling. Sci. Rep. 9 (1), 15778. 10.1038/s41598-019-52215-4 31673015 PMC6823391

[B70] Lancaster M. A. Renner M. Martin C.-A. Wenzel D. Bicknell L. S. Hurles M. E. (2013). Cerebral organoids model human brain development and microcephaly. Nature 501 (7467), 373–379. 10.1038/nature12517 23995685 PMC3817409

[B71] Lee M.-W. Lee W.-H. Ryu C.-K. Lee Y.-M. Lee Y.-H. Kim J.-Y. (2021). Peripapillary retinal nerve fiber layer and microvasculature in prolonged type 2 diabetes patients without clinical diabetic retinopathy. Investigative Ophthalmol. & Vis. Sci. 62 (2), 9. 10.1167/iovs.62.2.9 PMC7873502 33733716

[B212] Lei Y. Tang R. Xu J. Wang W. Zhang B. Liu J. (2021). Applications of single-cell sequencing in cancer research: progress and perspectives. J. Hematol. Oncol. 14 (1), 91. 10.1186/s13045-021-01105-2 34108022 PMC8190846

[B72] Li L. Bowling S. McGeary S. E. Yu Q. Lemke B. Alcedo K. (2023). A mouse model with high clonal barcode diversity for joint lineage, transcriptomic, and epigenomic profiling in single cells. Cell 186 (23), 5183–5199.e22. 10.1016/j.cell.2023.09.019 37852258 PMC13409469

[B73] Li X. Yu Z.-W. Li H.-Y. Yuan Y. Gao X.-Y. Kuang H.-Y. (2021). Retinal microglia polarization in diabetic retinopathy. Vis. Neurosci. 38, E006. 10.1017/S0952523821000031 33934736

[B74] Liao D. Fan W. Li N. Li R. Wang X. Liu J. (2024). A single cell atlas of circulating immune cells involved in diabetic retinopathy. IScience 27 (2), 109003. 10.1016/j.isci.2024.109003 38327792 PMC10847734

[B75] Lin P. Gan Y.-B. He J. Lin S.-E. Xu J.-K. Chang L. (2024). Advancing skeletal health and disease research with single-cell RNA sequencing. Mil. Med. Res. 11 (1), 33. 10.1186/s40779-024-00538-3 38816888 PMC11138034

[B76] Lin T.-Y. Kang E. Y.-C. Shao S.-C. Lai E. C.-C. Garg S. J. Chen K.-J. (2023). Risk of diabetic retinopathy between sodium-glucose cotransporter-2 inhibitors and glucagon-like peptide-1 receptor agonists. Diabetes & Metabolism J. 47 (3), 394–404. 10.4093/dmj.2022.0221 PMC10244193 36872060

[B77] Liu H. Stepicheva N. A. Ghosh S. Shang P. Chowdhury O. Daley R. A. (2022a). Reducing Akt2 in retinal pigment epithelial cells causes a compensatory increase in Akt1 and attenuates diabetic retinopathy. Nat. Commun. 13 (1), 6045. 10.1038/s41467-022-33773-0 36229454 PMC9561713

[B78] Liu J. Liao X. Li N. Xu Z. Yang W. Zhou H. (2024). MedComm 5 (4), e534. [Not Available]. 10.1002/mco2.534 38585235 PMC10999176

[B79] Liu X. Luo T. Fan Z. Li J. Zhang Y. Lu G. (2023). Single cell RNA-seq resolution revealed CCR1+/SELL+/XAF+ CD14 monocytes mediated vascular endothelial cell injuries in Kawasaki disease and COVID-19. Biochimica Biophysica Acta. Mol. Basis Dis. 1869 (5), 166707. 10.1016/j.bbadis.2023.166707 PMC10052884 37001702

[B80] Liu Z. Shi H. Xu J. Yang Q. Ma Q. Mao X. (2022b). Single-cell transcriptome analyses reveal microglia types associated with proliferative retinopathy. JCI Insight 7 (23), e160940. 10.1172/jci.insight.160940 36264636 PMC9746914

[B81] Li W W. Lou X. Zha Y. Qin Y. Zha J. Hong L. (2023). Single-cell RNA-seq of heart reveals intercellular communication drivers of myocardial fibrosis in diabetic cardiomyopathy. ELife 12, e80479. 10.7554/eLife.80479 37010266 PMC10110238

[B82] Llorian-Salvador M. Cabeza-Fernandez S. Gomez-Sanchez J. A. de la Fuente A. G. (2024). Glial cell alterations in diabetes-induced neurodegeneration. Cell Mol. Life Sci. 81 (1), 47. 10.1007/s00018-023-05024-y 38236305 PMC10796438

[B83] Lou B. Zeng L. Gao X. Qian X. Li J. J. Gu X. (2022). A single-cell transcriptomic atlas of the human ciliary body. Cell. Mol. Life Sci. CMLS 79 (10), 528. 10.1007/s00018-022-04559-w 36163311 PMC9512889

[B84] Lukowski S. W. Lo C. Y. Sharov A. A. Nguyen Q. Fang L. Hung S. S. (2019). A single-cell transcriptome atlas of the adult human retina. EMBO J. 38 (18), e100811. 10.15252/embj.2018100811 31436334 PMC6745503

[B85] Luo J. Deng M. Zhang X. Sun X. (2023). ESICCC as a systematic computational framework for evaluation, selection, and integration of cell-cell communication inference methods. Genome Res. 33 (10), 1788–1805. 10.1101/gr.278001.123 37827697 PMC10691505

[B86] Lv K. Ying H. Hu G. Hu J. Jian Q. Zhang F. (2022). Integrated multi-omics reveals the activated retinal microglia with intracellular metabolic reprogramming contributes to inflammation in STZ-induced early diabetic retinopathy. Front. Immunol. 13, 942768. 10.3389/fimmu.2022.942768 36119084 PMC9479211

[B87] Lv Z Z. Han J. Li J. Guo H. Fei Y. Sun Z. (2022). Single cell RNA-seq analysis identifies ferroptotic chondrocyte cluster and reveals TRPV1 as an anti-ferroptotic target in osteoarthritis. EBioMedicine 84, 104258. 10.1016/j.ebiom.2022.104258 36137413 PMC9494174

[B88] Ma P. Amemiya H. M. He L. L. Gandhi S. J. Nicol R. Bhattacharyya R. P. (2023). Bacterial droplet-based single-cell RNA-seq reveals antibiotic-associated heterogeneous cellular states. Cell 186 (4), 877–891.e14. 10.1016/j.cell.2023.01.002 36708705 PMC10014032

[B89] Manochkumar J. Cherukuri A. K. Kumar R. S. Almansour A. I. Ramamoorthy S. Efferth T. (2023). A critical review of machine-learning for “multi-omics” marine metabolite datasets. Comput. Biol. Med. 165, 107425. 10.1016/j.compbiomed.2023.107425 37696182

[B90] Mao P. Shen Y. Mao X. Liu K. Zhong J. (2023). The single-cell landscape of alternative transcription start sites of diabetic retina. Exp. Eye Res. 233, 109520. 10.1016/j.exer.2023.109520 37236522

[B91] Mao S. Wang Y. Chao N. Zeng L. Zhang L. (2024). Integrated analysis of single-cell RNA-seq and bulk RNA-seq reveals immune suppression subtypes and establishes a novel signature for determining the prognosis in lung adenocarcinoma. Cell. Oncol. Dordr. 47, 1697–1713. 10.1007/s13402-024-00948-4 38616208 PMC12974075

[B92] Marneros A. G. (2023). Role of inflammasome activation in neovascular age-related macular degeneration. FEBS J. 290 (1), 28–36. 10.1111/febs.16278 34767301 PMC9185667

[B93] Matosin N. Arloth J. Czamara D. Edmond K. Z. Maitra M. Fröhlich A. S. (2023). Associations of psychiatric disease and ageing with FKBP5 expression converge on superficial layer neurons of the neocortex. Acta Neuropathol. 145 (4), 439–459. 10.1007/s00401-023-02541-9 36729133 PMC10020280

[B94] McIntosh T. Walsh H. Baldwin K. Iltis A. Mohan S. Sawinski D. (2024). Evaluating ApoL1 genetic testing policy options for transplant centers: a delphi consensus panel project with stakeholders. Clin. J. Am. Soc. Nephrol. CJASN 19 (4), 494–502. 10.2215/CJN.0000000000000397 38190141 PMC11020447

[B95] McNulty R. Sritharan D. Pahng S. H. Meisch J. P. Liu S. Brennan M. A. (2023). Probe-based bacterial single-cell RNA sequencing predicts toxin regulation. Nat. Microbiol. 8 (5), 934–945. 10.1038/s41564-023-01348-4 37012420 PMC10159851

[B96] Md Pauzi S. H. Masir N. Yahaya A. Mohammed F. Tizen Laim N. M. S. Mustangin M. (2021). HER2 testing by immunohistochemistry in breast cancer: a multicenter proficiency ring study. Indian J. Pathology & Microbiol. 64 (4), 677–682. 10.4103/IJPM.IJPM_983_20 34673585

[B97] Motloch K. Soler V. Delyfer M.-N. Vasseur V. Wolff B. Issa M. (2024). Efficacy and safety of 0.19-mg fluocinolone acetonide implant in postoperative cystoid macular edema after pars plana vitrectomy: the ILUvien in Postoperative CYstoid macular eDema study. Ophthalmol. Retina. 10.1016/j.oret.2024.07.004 39004282

[B98] Mullin N. K. Voigt A. P. Flamme-Wiese M. J. Liu X. Riker M. J. Varzavand K. (2023). Multimodal single-cell analysis of nonrandom heteroplasmy distribution in human retinal mitochondrial disease. JCI Insight 8 (14), e165937. 10.1172/jci.insight.165937 37289546 PMC10443808

[B99] Netea M. G. Domínguez-Andrés J. Barreiro L. B. Chavakis T. Divangahi M. Fuchs E. (2020). Defining trained immunity and its role in health and disease. Nat. Rev. Immunol. 20 (6), 375–388. 10.1038/s41577-020-0285-6 32132681 PMC7186935

[B100] Nishikawa H. Koyama S. (2021). Mechanisms of regulatory T cell infiltration in tumors: implications for innovative immune precision therapies. J. For Immunother. Cancer 9 (7), e002591. 10.1136/jitc-2021-002591 PMC8327843 34330764

[B101] Niu T. Fang J. Shi X. Zhao M. Xing X. Wang Y. (2021). Pathogenesis study based on high-throughput single-cell sequencing analysis reveals novel transcriptional landscape and heterogeneity of retinal cells in type 2 diabetic mice. Diabetes 70 (5), 1185–1197. 10.2337/db20-0839 33674409

[B102] Nouri H. Abtahi S.-H. Mazloumi M. Samadikhadem S. Arevalo J. F. Ahmadieh H. (2024). Optical coherence tomography angiography in diabetic retinopathy: a major review. Surv. Ophthalmol. 69 (4), 558–574. 10.1016/j.survophthal.2024.03.004 38521424

[B103] Paik D. T. Cho S. Tian L. Chang H. Y. Wu J. C. (2020). Single-cell RNA sequencing in cardiovascular development, disease and medicine. Nat. Rev. Cardiol. 17 (8), 457–473. 10.1038/s41569-020-0359-y 32231331 PMC7528042

[B104] Parikh B. H. Blakeley P. Regha K. Liu Z. Yang B. Bhargava M. (2023). Single-cell transcriptomics reveals maturation of transplanted stem cell-derived retinal pigment epithelial cells toward native state. Proc. Natl. Acad. Sci. U. S. A. 120 (26), e2214842120. 10.1073/pnas.2214842120 37339216 PMC10293804

[B105] Pei W. Shang F. Wang X. Fanti A.-K. Greco A. Busch K. (2020). Resolving fates and single-cell transcriptomes of hematopoietic stem cell clones by PolyloxExpress barcoding. Cell Stem Cell 27 (3), 383–395. 10.1016/j.stem.2020.07.018 32783885

[B106] Pelka K. Hofree M. Chen J. H. Sarkizova S. Pirl J. D. Jorgji V. (2021). Spatially organized multicellular immune hubs in human colorectal cancer. Cell 184 (18), 4734–4752.e20. 10.1016/j.cell.2021.08.003 34450029 PMC8772395

[B107] Peng G. Cui G. Ke J. Jing N. (2020). Using single-cell and spatial transcriptomes to understand stem cell lineage specification during early embryo development. Annu. Rev. Genomics Hum. Genet. 21, 163–181. 10.1146/annurev-genom-120219-083220 32339035

[B108] Peng H. Li H. Ma B. Sun X. Chen B. (2024). DJ-1 regulates mitochondrial function and promotes retinal ganglion cell survival under high glucose-induced oxidative stress. Front. Pharmacol. 15, 1455439. 10.3389/fphar.2024.1455439 39323632 PMC11422208

[B109] Peng W. Gutierrez Reyes C. D. Gautam S. Yu A. Cho B. G. Goli M. (2023). MS-based glycomics and glycoproteomics methods enabling isomeric characterization. Mass Spectrom. Rev. 42 (2), 577–616. 10.1002/mas.21713 34159615 PMC8692493

[B110] Perais J. Agarwal R. Evans J. R. Loveman E. Colquitt J. L. Owens D. (2023). Prognostic factors for the development and progression of proliferative diabetic retinopathy in people with diabetic retinopathy. Cochrane Database Syst. Rev. 2 (2), CD013775. 10.1002/14651858.CD013775.pub2 36815723 PMC9943918

[B111] Pfeifer C. W. Walsh J. T. Santeford A. Lin J. B. Beatty W. L. Terao R. (2023). Dysregulated CD200-CD200R signaling in early diabetes modulates microglia-mediated retinopathy. Proc. Natl. Acad. Sci. U. S. A. 120 (45), e2308214120. 10.1073/pnas.2308214120 37903272 PMC10636339

[B112] Plumbly W. Brandon N. Deeb T. Z. Hall J. Harwood A. J. (2019). L-type voltage-gated calcium channel regulation of *in vitro* human cortical neuronal networks. Sci. Rep. 9 (1), 13810. 10.1038/s41598-019-50226-9 31554851 PMC6761148

[B113] Pratama S. Lauren B. C. Wisnu W. (2022). The efficacy of vitamin B12 supplementation for treating vitamin B12 deficiency and peripheral neuropathy in metformin-treated type 2 diabetes mellitus patients: a systematic review. Diabetes & Metabolic Syndrome 16 (10), 102634. 10.1016/j.dsx.2022.102634 36240684

[B114] Pun M. Pratt D. Nano P. R. Joshi P. K. Jiang L. Englinger B. (2023). Common molecular features of H3K27M DMGs and PFA ependymomas map to hindbrain developmental pathways. Acta Neuropathol. Commun. 11 (1), 25. 10.1186/s40478-023-01514-z 36759899 PMC9912509

[B115] Qin G. B. Wu Y. H. Chen H. S. Huang Y. T. Yi J. F. Xiao Y. (2023). Correlation analysis between morphologic characteristics of the thoracolumbar basivertebral foramen and Kummell's disease in patients with osteoporosis using imaging techniques. BMC Musculoskelet. Disord. 24 (1), 513. 10.1186/s12891-023-06609-1 37353769 PMC10288774

[B116] Quadrato G. Nguyen T. Macosko E. Z. Sherwood J. L. Min Yang S. Berger D. R. (2017). Cell diversity and network dynamics in photosensitive human brain organoids. Nature 545 (7652), 48–53. 10.1038/nature22047 28445462 PMC5659341

[B117] Raj B. Gagnon J. A. Schier A. F. (2018a). Large-scale reconstruction of cell lineages using single-cell readout of transcriptomes and CRISPR-Cas9 barcodes by scGESTALT. Nat. Protoc. 13 (11), 2685–2713. 10.1038/s41596-018-0058-x 30353175 PMC6279253

[B118] Raj B. Wagner D. E. McKenna A. Pandey S. Klein A. M. Shendure J. (2018b). Simultaneous single-cell profiling of lineages and cell types in the vertebrate brain. Nat. Biotechnol. 36 (5), 442–450. 10.1038/nbt.4103 29608178 PMC5938111

[B119] Rangasamy S. Monickaraj F. Legendre C. Cabrera A. P. Llaci L. Bilagody C. (2020). Transcriptomics analysis of pericytes from retinas of diabetic animals reveals novel genes and molecular pathways relevant to blood-retinal barrier alterations in diabetic retinopathy. Exp. Eye Res. 195, 108043. 10.1016/j.exer.2020.108043 32376470 PMC7323486

[B120] Rauscher F. G. Elze T. Francke M. Martinez-Perez M. E. Li Y. Wirkner K. (2024). Glucose tolerance and insulin resistance/sensitivity associate with retinal layer characteristics: the LIFE-Adult-Study. Diabetologia 67 (5), 928–939. 10.1007/s00125-024-06093-9 38431705 PMC10954961

[B121] Ren H. Zhang Y. Zhong M. Hussian J. Tang Y. Liu S. (2023). Calcium signaling-mediated transcriptional reprogramming during abiotic stress response in plants. TAG. Theor. Appl. Genet. Theor. Und Angewandte Genet. 136 (10), 210. 10.1007/s00122-023-04455-2 37728763

[B122] Ren L. Xia J. Huang C. Bai Y. Yao J. Li D. (2024). Single-cell transcriptomic analysis reveals the antiangiogenic role of Mgarp in diabetic retinopathy. BMJ Open Diabetes Res. & Care 12 (4), e004189. 10.1136/bmjdrc-2024-004189 PMC11268071 39013633

[B123] Replogle J. M. Norman T. M. Xu A. Hussmann J. A. Chen J. Cogan J. Z. (2020). Combinatorial single-cell CRISPR screens by direct guide RNA capture and targeted sequencing. Nat. Biotechnol. 38 (8), 954–961. 10.1038/s41587-020-0470-y 32231336 PMC7416462

[B124] Rohlenova K. Goveia J. García-Caballero M. Subramanian A. Kalucka J. Treps L. (2020). Single-cell RNA sequencing maps endothelial metabolic plasticity in pathological angiogenesis. Cell Metab. 31 (4), 862–877. 10.1016/j.cmet.2020.03.009 32268117

[B125] Saddala M. S. Mundla S. Patyal N. Dash S. (2023). Single-cell RNA sequencing (scRNA-Seq) data analysis of retinal homeostasis and degeneration of microglia. Methods Mol. Biol. 2678, 91–106. 10.1007/978-1-0716-3255-0_6 37326706

[B126] Saint-Antoine M. M. Singh A. (2020). Network inference in systems biology: recent developments, challenges, and applications. Curr. Opin. Biotechnol. 63, 89–98. 10.1016/j.copbio.2019.12.002 31927423 PMC7308210

[B127] SamantaP.CookeS. F.McNultyR.HormozS.RosenthalA. (2024). ProBac-seq, a bacterial single-cell RNA sequencing methodology using droplet microfluidics and large oligonucleotide probe sets. Nat. Protoc. 19, 2939–2966. 10.1038/s41596-024-01002-1 38769144

[B128] Shekhar K. Lapan S. W. Whitney I. E. Tran N. M. Macosko E. Z. Kowalczyk M. (2016). Comprehensive classification of retinal bipolar neurons by single-cell transcriptomics. Cell 166 (5), 1308–1323. 10.1016/j.cell.2016.07.054 27565351 PMC5003425

[B129] Shi S. Ding C. Zhu S. Xia F. Buscho S. E. Li S. (2023). PERK inhibition suppresses neovascularization and protects neurons during ischemia-induced retinopathy. Investigative Ophthalmol. & Vis. Sci. 64 (11), 17. 10.1167/iovs.64.11.17 PMC10424802 37566408

[B130] Singer M. A. Sheth V. Mansour S. E. Coughlin B. Gonzalez V. H. (2022). Three-year safety and efficacy of the 0.19-mg fluocinolone acetonide intravitreal implant for diabetic macular edema: the PALADIN study. Ophthalmology 129 (6), 605–613. 10.1016/j.ophtha.2022.01.015 35063472

[B131] Skol A. D. Jung S. C. Sokovic A. M. Chen S. Fazal S. Sosina O. (2020). Integration of genomics and transcriptomics predicts diabetic retinopathy susceptibility genes. ELife 9, e59980. 10.7554/eLife.59980 33164750 PMC7728435

[B132] Soni D. Sagar P. Takkar B. (2021). Diabetic retinal neurodegeneration as a form of diabetic retinopathy. Int. Ophthalmol. 41 (9), 3223–3248. 10.1007/s10792-021-01864-4 33954860

[B133] Sørensen C. B. Adams T. B. Pedersen E. R. Nielsen J. Schmidt J. H. (2023). AMTASTM and user-operated smartphone research application audiometry-An evaluation study. PLoS One 18 (9), e0291412. 10.1371/journal.pone.0291412 37708125 PMC10501612

[B134] Spencer B. G. Estevez J. J. Liu E. Craig J. E. Finnie J. W. (2020). Pericytes, inflammation, and diabetic retinopathy. Inflammopharmacology 28 (3), 697–709. 10.1007/s10787-019-00647-9 31612299

[B135] Starr C. R. Zhylkibayev A. Mobley J. A. Gorbatyuk M. S. (2023). Proteomic analysis of diabetic retinas. Front. Endocrinol. 14, 1229089. 10.3389/fendo.2023.1229089 PMC10486886 37693346

[B136] Stuart T. Butler A. Hoffman P. Hafemeister C. Papalexi E. Mauck W. M. (2019). Comprehensive integration of single-cell data. Cell 177 (7), 1888–1902. 10.1016/j.cell.2019.05.031 31178118 PMC6687398

[B137] Sun F. Li H. Sun D. Fu S. Gu L. Shao X. (2024). Single-cell omics: experimental workflow, data analyses and applications. Sci. China. Life Sci. 10.1007/s11427-023-2561-0 39060615

[B138] Sun L. Wang R. Hu G. Liu H. Lv K. Duan Y. (2021). Single cell RNA sequencing (scRNA-Seq) deciphering pathological alterations in streptozotocin-induced diabetic retinas. Exp. Eye Res. 210, 108718. 10.1016/j.exer.2021.108718 34364890

[B139] Tan A. Li T. Ruan L. Yang J. Luo Y. Li L. (2021). Knockdown of Malat1 alleviates high-glucose-induced angiogenesis through regulating miR-205-5p/VEGF-A axis. Exp. Eye Res. 207, 108585. 10.1016/j.exer.2021.108585 33887222

[B140] Tan T.-E. Wong T. Y. (2022). Diabetic retinopathy: looking forward to 2030. Front. Endocrinol. 13, 1077669. 10.3389/fendo.2022.1077669 PMC9868457 36699020

[B141] Tang L. Xu G.-T. Zhang J.-F. (2023). Inflammation in diabetic retinopathy: possible roles in pathogenesis and potential implications for therapy. Neural Regen. Res. 18 (5), 976–982. 10.4103/1673-5374.355743 36254977 PMC9827774

[B142] Tan Y Y. Huang J. Li D. Zou C. Liu D. Qin B. (2023). Single-cell RNA sequencing in dissecting microenvironment of age-related macular degeneration: challenges and perspectives. Ageing Res. Rev. 90, 102030. 10.1016/j.arr.2023.102030 37549871

[B143] Tan Z Z. Chen X. Zuo J. Fu S. Wang J. Wang H. (2023). Integrating bulk and single-cell RNA sequencing reveals heterogeneity, tumor microenvironment, and immunotherapeutic efficacy based on sialylation-related genes in bladder cancer. J. Inflamm. Res. 16, 3399–3417. 10.2147/JIR.S418433 37600224 PMC10438438

[B214] Temple S. (2023). Advancing cell therapy for neurodegenerative diseases. Cell Stem Cell 30 (5), 512–529. 10.1016/j.stem.2023.03.017 37084729 PMC10201979

[B144] Tolentino M. J. Tolentino A. J. Tolentino E. M. Krishnan A. Genead M. A. (2023). Sialic acid mimetic microglial sialic acid-binding immunoglobulin-like lectin agonism: potential to restore retinal homeostasis and regain visual function in age-related macular degeneration. Pharm. Basel, Switz. 16 (12), 1735. 10.3390/ph16121735 PMC10747662 38139861

[B145] Tresenrider A. Sridhar A. Eldred K. C. Cuschieri S. Hoffer D. Trapnell C. (2023). Single-cell sequencing of individual retinal organoids reveals determinants of cell-fate heterogeneity. Cell Rep. Methods 3 (8), 100548. 10.1016/j.crmeth.2023.100548 37671011 PMC10475847

[B146] Uemura A. Fruttiger M. D'Amore P. A. De Falco S. Joussen A. M. Sennlaub F. (2021). VEGFR1 signaling in retinal angiogenesis and microinflammation. Prog. Retin Eye Res. 84, 100954. 10.1016/j.preteyeres.2021.100954 33640465 PMC8385046

[B147] Valecha M. Posada D. (2022). Somatic variant calling from single-cell DNA sequencing data. Comput. Struct. Biotechnol. J. 20, 2978–2985. 10.1016/j.csbj.2022.06.013 35782734 PMC9218383

[B148] Van Bergen T. Etienne I. Cunningham F. Moons L. Schlingemann R. O. Feyen J. H. M. (2019). The role of placental growth factor (PlGF) and its receptor system in retinal vascular diseases. Prog. Retin. Eye Res. 69, 116–136. 10.1016/j.preteyeres.2018.10.006 30385175

[B149] Van de Sande B. Lee J. S. Mutasa-Gottgens E. Naughton B. Bacon W. Manning J. (2023). Applications of single-cell RNA sequencing in drug discovery and development. Nat. Rev. Drug Discov. 22 (6), 496–520. 10.1038/s41573-023-00688-4 37117846 PMC10141847

[B150] VanHorn S. Morris S. A. (2021). Next-generation lineage tracing and fate mapping to interrogate development. Dev. Cell 56 (1), 7–21. 10.1016/j.devcel.2020.10.021 33217333

[B151] Van Hove I. De Groef L. Boeckx B. Modave E. Hu T.-T. Beets K. (2020). Single-cell transcriptome analysis of the Akimba mouse retina reveals cell-type-specific insights into the pathobiology of diabetic retinopathy. Diabetologia 63 (10), 2235–2248. 10.1007/s00125-020-05218-0 32734440

[B152] Varughese G. I. Jacob S. (2023). Sodium-glucose co-transporter 2 inhibitors and glucagon-like peptide 1 receptor agonists treatment: variable observations of sequelae on diabetic retinopathy. J. R. Soc. Med. 116 (12), 408. 10.1177/01410768231215999 38054392 PMC10850872

[B153] Voight B. F. Scott L. J. Steinthorsdottir V. Morris A. P. Dina C. Welch R. P. (2010). Twelve type 2 diabetes susceptibility loci identified through large-scale association analysis. Nat. Genet. 42 (7), 579–589. 10.1038/ng.609 20581827 PMC3080658

[B154] Voigt A. P. Mullin N. K. Navratil E. M. Flamme-Wiese M. J. Lin L.-C. Scheetz T. E. (2023). Gene expression within a human choroidal neovascular membrane using spatial transcriptomics. Investigative Ophthalmol. & Vis. Sci. 64 (13), 40. 10.1167/iovs.64.13.40 PMC10615143 37878301

[B155] Voigt A. P. Mullin N. K. Stone E. M. Tucker B. A. Scheetz T. E. Mullins R. F. (2021a). Single-cell RNA sequencing in vision research: insights into human retinal health and disease. Prog. Retin Eye Res. 83, 100934. 10.1016/j.preteyeres.2020.100934 33383180 PMC8236499

[B156] Voigt A. P. Mullin N. K. Whitmore S. S. DeLuca A. P. Burnight E. R. Liu X. (2021b). Human photoreceptor cells from different macular subregions have distinct transcriptional profiles. Hum. Mol. Genet. 30 (16), 1543–1558. 10.1093/hmg/ddab140 34014299 PMC8330894

[B157] Voigt A. P. Whitmore S. S. Flamme-Wiese M. J. Riker M. J. Wiley L. A. Tucker B. A. (2019). Molecular characterization of foveal versus peripheral human retina by single-cell RNA sequencing. Exp. Eye Res. 184, 234–242. 10.1016/j.exer.2019.05.001 31075224 PMC6596422

[B158] Vu T. N. Wills Q. F. Kalari K. R. Niu N. Wang L. Rantalainen M. (2016). Beta-Poisson model for single-cell RNA-seq data analyses. Bioinforma. Oxf. Engl. 32 (14), 2128–2135. 10.1093/bioinformatics/btw202 PMC13048230 27153638

[B159] Wang D. He P. Wang Z. Li G. Majed N. Gu A. Z. (2020). Advances in single cell Raman spectroscopy technologies for biological and environmental applications. Curr. Opin. Biotechnol. 64, 218–229. 10.1016/j.copbio.2020.06.011 32688195

[B160] Wang J. Sun H. Mou L. Lu Y. Wu Z. Pu Z. (2024). Unveiling the molecular complexity of proliferative diabetic retinopathy through scRNA-seq, AlphaFold 2, and machine learning. Front. Endocrinol. 15, 1382896. 10.3389/fendo.2024.1382896 PMC11116564 38800474

[B161] Wang J.-H. Kumar S. Liu G.-S. (2021). Bulk gene expression deconvolution reveals infiltration of M2 macrophages in retinal neovascularization. Investigative Ophthalmol. & Vis. Sci. 62 (14), 22. 10.1167/iovs.62.14.22 PMC8606818 34797904

[B162] Wang R. Yang X. Chen J. Zhang L. Griffiths J. A. Cui G. (2023). Time space and single-cell resolved tissue lineage trajectories and laterality of body plan at gastrulation. Nat. Commun. 14 (1), 5675. 10.1038/s41467-023-41482-5 37709743 PMC10502153

[B163] Wang C C. Yu Q. Song T. Wang Z. Song L. Yang Y. (2022). The heterogeneous immune landscape between lung adenocarcinoma and squamous carcinoma revealed by single-cell RNA sequencing. Signal Transduct. Target. Ther. 7 (1), 289. 10.1038/s41392-022-01130-8 36008393 PMC9411197

[B164] Wang N N. Wei L. Liu D. Zhang Q. Xia X. Ding L. (2022). Identification and validation of autophagy-related genes in diabetic retinopathy. Front. Endocrinol. 13, 867600. 10.3389/fendo.2022.867600 PMC9098829 35574010

[B165] Wang Y Y. Yang X. Li Q. Zhang Y. Chen L. Hong L. (2022). Single-cell RNA sequencing reveals the Müller subtypes and inner blood-retinal barrier regulatory network in early diabetic retinopathy. Front. Mol. Neurosci. 15, 1048634. 10.3389/fnmol.2022.1048634 36533134 PMC9754943

[B166] Wang Y Y. Yang X. Zhang Y. Hong L. Xie Z. Jiang W. (2024). Single-cell RNA sequencing reveals roles of unique retinal microglia types in early diabetic retinopathy. Diabetology & Metabolic Syndrome 16 (1), 49. 10.1186/s13098-024-01282-3 38409074 PMC10895757

[B167] Weber L. L. Sashittal P. El-Kebir M. (2021). doubletD: detecting doublets in single-cell DNA sequencing data. Bioinforma. Oxf. Engl. 37 (Suppl. l_1), i214–i221. 10.1093/bioinformatics/btab266 PMC8275324 34252961

[B168] Wimmers F. Donato M. Kuo A. Ashuach T. Gupta S. Li C. (2021). The single-cell epigenomic and transcriptional landscape of immunity to influenza vaccination. Cell 184 (15), 3915–3935.e21. 10.1016/j.cell.2021.05.039 34174187 PMC8316438

[B169] Wolf J. Rasmussen D. K. Sun Y. J. Vu J. T. Wang E. Espinosa C. (2023). Liquid-biopsy proteomics combined with AI identifies cellular drivers of eye aging and disease *in vivo* . Cell 186 (22), 4868–4884.e12. 10.1016/j.cell.2023.09.012 37863056 PMC10720485

[B170] Wu H. Wang M. Li X. Shao Y. (2021). The metaflammatory and immunometabolic role of macrophages and microglia in diabetic retinopathy. Hum. Cell 34 (6), 1617–1628. 10.1007/s13577-021-00580-6 34324139

[B171] Wurl J. A. Mac Nair C. E. Dietz J. A. Shestopalov V. I. Nickells R. W. (2023). Contralateral astrocyte response to acute optic nerve damage is mitigated by PANX1 channel activity. Int. J. Mol. Sci. 24 (21), 15641. 10.3390/ijms242115641 37958624 PMC10647301

[B172] Xia M. Jiao L. Wang X.-H. Tong M. Yao M.-D. Li X.-M. (2023). Single-cell RNA sequencing reveals a unique pericyte type associated with capillary dysfunction. Theranostics 13 (8), 2515–2530. 10.7150/thno.83532 37215579 PMC10196835

[B173] Xia Y. Luo Q. Chen J. Huang C. Jahangir A. Pan T. (2022). Retinal astrocytes and microglia activation in diabetic retinopathy rhesus monkey models. Curr. Eye Res. 47 (2), 297–303. 10.1080/02713683.2021.1984535 34547966

[B174] Xiao Y. Hu X. Fan S. Zhong J. Mo X. Liu X. (2021). Single-cell transcriptome profiling reveals the suppressive role of retinal neurons in microglia activation under diabetes mellitus. Front. Cell Dev. Biol. 9, 680947. 10.3389/fcell.2021.680947 34434927 PMC8381733

[B175] Xiong X. Kuang H. Ansari S. Liu T. Gong J. Wang S. (2019). Landscape of intercellular crosstalk in healthy and NASH liver revealed by single-cell secretome gene analysis. Mol. Cell 75 (3), 644–660. 10.1016/j.molcel.2019.07.028 31398325 PMC7262680

[B176] Xu C. Liu G. Liu X. Wang F. (2014). O-GlcNAcylation under hypoxic conditions and its effects on the blood-retinal barrier in diabetic retinopathy. Int. J. Mol. Med. 33 (3), 624–632. 10.3892/ijmm.2013.1597 24366041

[B177] Xu L. Chang C. Jiang P. Wei K. Zhang R. Jin Y. (2022). Metabolomics in rheumatoid arthritis: advances and review. Front. Immunol. 13, 961708. 10.3389/fimmu.2022.961708 36032122 PMC9404373

[B178] Xu X. Crow M. Rice B. R. Li F. Harris B. Liu L. (2021). Single-cell RNA sequencing of developing maize ears facilitates functional analysis and trait candidate gene discovery. Dev. Cell 56 (4), 557–568.e6. 10.1016/j.devcel.2020.12.015 33400914 PMC7904613

[B179] Xu X. Zhang C. Tang G. Wang N. Feng Y. (2023). Single-cell transcriptome profiling highlights the role of APP in blood vessels in assessing the risk of patients with proliferative diabetic retinopathy developing Alzheimer's disease. Front. Cell Dev. Biol. 11, 1328979. 10.3389/fcell.2023.1328979 38328307 PMC10847282

[B180] Xu Y. Huang S. Li Z. Dai L. Wu H. Wang P. (2023a). Single-cell RNA landscape of osteoimmune microenvironment in osteoporotic vertebral compression fracture and Kümmell's disease. Front. Cell Dev. Biol. 11, 1276098. 10.3389/fcell.2023.1276098 38161331 PMC10755405

[B181] Xu Y. Xiang Z. E W. Lang Y. Huang S. Qin W. (2023b). Single-cell transcriptomes reveal a molecular link between diabetic kidney and retinal lesions. Commun. Biol. 6 (1), 912. 10.1038/s42003-023-05300-4 37670124 PMC10480496

[B182] Xu Y. Zhang Y. Qin Y. Gu M. Chen R. Sun Y. (2023c). Multi-omics analysis of functional substances and expression verification in cashmere fineness. BMC Genomics 24 (1), 720. 10.1186/s12864-023-09825-0 38017403 PMC10685610

[B183] Yang C. Ma Y. Yao M. Jiang Q. Xue J. (2024). Causal relationships between blood metabolites and diabetic retinopathy: a two-sample Mendelian randomization study. Front. Endocrinol. 15, 1383035. 10.3389/fendo.2024.1383035 PMC11094203 38752182

[B184] Yang J. Liu Z. (2022). Mechanistic pathogenesis of endothelial dysfunction in diabetic nephropathy and retinopathy. Front. Endocrinol. 13, 816400. 10.3389/fendo.2022.816400 PMC9174994 35692405

[B185] Yang Y. Sun L. Chen Z. Liu W. Xu Q. Liu F. (2023). The immune-metabolic crosstalk between CD3+C1q+TAM and CD8+T cells associated with relapse-free survival in HCC. Front. Immunol. 14, 1033497. 10.3389/fimmu.2023.1033497 36845133 PMC9948089

[B186] Yang Z Z. Liu Y. Chen X. Huang S. Li Y. Ye G. (2023). Empagliflozin targets Mfn1 and Opa1 to attenuate microglia-mediated neuroinflammation in retinal ischemia and reperfusion injury. J. Neuroinflammation 20 (1), 296. 10.1186/s12974-023-02982-9 38082266 PMC10714482

[B187] Yao M. Ren T. Pan Y. Xue X. Li R. Zhang L. (2022). A new generation of lineage tracing dynamically records cell fate choices. Int. J. Mol. Sci. 23 (9), 5021. 10.3390/ijms23095021 35563412 PMC9105840

[B188] Yao X. Zhao Z. Zhang W. Liu R. Ni T. Cui B. (2024). Specialized retinal endothelial cells modulate blood-retina barrier in diabetic retinopathy. Diabetes 73 (2), 225–236. 10.2337/db23-0368 37976214

[B189] Yao Y. Chen Z. Wu Q. Lu Y. Zhou X. Zhu X. (2023). Single-cell RNA sequencing of retina revealed novel transcriptional landscape in high myopia and underlying cell-type-specific mechanisms. MedComm 4 (5), e372. 10.1002/mco2.372 37746666 PMC10511833

[B190] Ye C. Chen Z. Liu Z. Wang F. He X. (2020). Defining endogenous barcoding sites for CRISPR/Cas9-based cell lineage tracing in zebrafish. J. Genet. Genomics = Yi Chuan Xue Bao 47 (2), 85–91. 10.1016/j.jgg.2019.11.012 32173285

[B191] Yeh C.-F. Chen Y.-H. Liu S.-F. Kao H.-L. Wu M.-S. Yang K.-C. (2020). Mutual interplay of host immune system and gut microbiota in the immunopathology of atherosclerosis. Int. J. Mol. Sci. 21 (22), 8729. 10.3390/ijms21228729 33227973 PMC7699263

[B192] Yi W. Lu Y. Zhong S. Zhang M. Sun L. Dong H. (2021). A single-cell transcriptome atlas of the aging human and macaque retina. Natl. Sci. Rev. 8 (4), nwaa179. 10.1093/nsr/nwaa179 34691611 PMC8288367

[B193] Yousri N. A. Suhre K. Yassin E. Al-Shakaki A. Robay A. Elshafei M. (2022). Metabolic and metabo-clinical signatures of type 2 diabetes, obesity, retinopathy, and dyslipidemia. Diabetes 71 (2), 184–205. 10.2337/db21-0490 34732537 PMC8914294

[B194] Yu L. Shen N. Shi Y. Shi X. Fu X. Li S. (2022). Characterization of cancer-related fibroblasts (CAF) in hepatocellular carcinoma and construction of CAF-based risk signature based on single-cell RNA-seq and bulk RNA-seq data. Front. Immunol. 13, 1009789. 10.3389/fimmu.2022.1009789 36211448 PMC9537943

[B195] Yuan D. Xu Y. Xue L. Zhang W. Gu L. Liu Q. (2024). Resveratrol protects against diabetic retinal ganglion cell damage by activating the Nrf2 signaling pathway. Heliyon 10 (9), e30786. 10.1016/j.heliyon.2024.e30786 38774075 PMC11107105

[B196] Zelniker T. A. Wiviott S. D. Raz I. Im K. Goodrich E. L. Furtado R. H. M. (2019). Comparison of the effects of glucagon-like peptide receptor agonists and sodium-glucose cotransporter 2 inhibitors for prevention of major adverse cardiovascular and renal outcomes in type 2 diabetes mellitus. Circulation 139 (17), 2022–2031. 10.1161/CIRCULATIONAHA.118.038868 30786725

[B197] Zhan L. (2023). Frontiers in understanding the pathological mechanism of diabetic retinopathy. Med. Sci. Monit. Int. Med. J. Exp. Clin. Res. 29, e939658. 10.12659/MSM.939658 PMC10274216 37307243

[B198] Zhang C. Zhao L. (2016). Strain-level dissection of the contribution of the gut microbiome to human metabolic disease. Genome Med. 8 (1), 41. 10.1186/s13073-016-0304-1 27098841 PMC4839137

[B199] Zhang J. Liu R. Kuang H.-Y. Gao X.-Y. Liu H.-L. (2017). Protective treatments and their target retinal ganglion cells in diabetic retinopathy. Brain Res. Bull. 132, 53–60. 10.1016/j.brainresbull.2017.05.007 28529157

[B200] Zhang J. Zhang J. Zhang C. Zhang J. Gu L. Luo D. (2022). Diabetic macular edema: current understanding, molecular mechanisms and therapeutic implications. Cells 11 (21), 3362. 10.3390/cells11213362 36359761 PMC9655436

[B201] Zhang P. Liu J. Pei S. Wu D. Xie J. Liu J. (2023). Mast cell marker gene signature: prognosis and immunotherapy response prediction in lung adenocarcinoma through integrated scRNA-seq and bulk RNA-seq. Front. Immunol. 14, 1189520. 10.3389/fimmu.2023.1189520 37256127 PMC10225553

[B202] Zhang Q. He Y. Luo N. Patel S. J. Han Y. Gao R. (2019). Landscape and dynamics of single immune cells in hepatocellular carcinoma. Cell 179 (4), 829–845. 10.1016/j.cell.2019.10.003 31675496

[B203] Zhang R. Huang C. Chen Y. Li T. Pang L. (2022). Single-cell transcriptomic analysis revealing changes in retinal cell subpopulation levels and the pathways involved in diabetic retinopathy. Ann. Transl. Med. 10 (10), 562. 10.21037/atm-22-1546 35722432 PMC9201190

[B204] Zhang X. Zhang F. Xu X. (2024). Single-cell RNA sequencing in exploring the pathogenesis of diabetic retinopathy. Clin. Transl. Med. 14 (7), e1751. 10.1002/ctm2.1751 38946005 PMC11214886

[B205] Zhang Y. Bailey T. S. Hittmeyer P. Dubois L. J. Theys J. Lambin P. (2024). Multiplex genetic manipulations in Clostridium butyricum and Clostridium sporogenes to secrete recombinant antigen proteins for oral-spore vaccination. Microb. Cell Factories 23 (1), 119. 10.1186/s12934-024-02389-y PMC11040787 38659027

[B206] Zhang Z. Cui F. Lin C. Zhao L. Wang C. Zou Q. (2021). Critical downstream analysis steps for single-cell RNA sequencing data. Briefings Bioinforma. 22 (5), bbab105. 10.1093/bib/bbab105 33822873

[B207] Zhang X X. Chao P. Zhang L. Xu L. Cui X. Wang S. (2023). Single-cell RNA and transcriptome sequencing profiles identify immune-associated key genes in the development of diabetic kidney disease. Front. Immunol. 14, 1030198. 10.3389/fimmu.2023.1030198 37063851 PMC10091903

[B208] Zhou H. Zhang L. Ding C. Zhou Y. Li Y. (2024). Upregulation of HMOX1 associated with M2 macrophage infiltration and ferroptosis in proliferative diabetic retinopathy. Int. Immunopharmacol. 134, 112231. 10.1016/j.intimp.2024.112231 38739977

[B209] Zhu H. Chen J. Liu K. Gao L. Wu H. Ma L. (2023). Human PBMC scRNA-seq-based aging clocks reveal ribosome to inflammation balance as a single-cell aging hallmark and super longevity. Sci. Adv. 9 (26), eabq7599. 10.1126/sciadv.abq7599 37379396 PMC10306289

[B210] Zhu L. Zhang D. Zhu H. Zhu J. Weng S. Dong L. (2018). Berberine treatment increases Akkermansia in the gut and improves high-fat diet-induced atherosclerosis in Apoe-/- mice. Atherosclerosis 268, 117–126. 10.1016/j.atherosclerosis.2017.11.023 29202334

